# 6-lncRNA Assessment Model for Monitoring and Prognosis of HER2-Positive Breast Cancer: Based on Transcriptome Data

**DOI:** 10.3389/pore.2021.609083

**Published:** 2021-04-13

**Authors:** Xiaoming Zhang, Haiyan Zhang, Jie Li, Xiaoran Ma, Zhengguo He, Cun Liu, Chundi Gao, Huayao Li, Xue Wang, Jibiao Wu

**Affiliations:** ^1^ College of Chinese Medicine, Shandong University of Traditional Chinese Medicine, Jinan, China; ^2^ The First Clinical Medical College, Shandong University of Traditional Chinese Medicine, Jinan, China; ^3^ Columbus Technical College, Columbus, GA, United States; ^4^ College of Basic Medicine, Qingdao University, Qingdao, China

**Keywords:** human epidermal growth factor receptor 2-positive breast cancer, random forests, prognosis, long non-coding RNA, competing endogenous RNA

## Abstract

**Background:** In view of the high malignancy and poor prognosis of human epidermal growth factor receptor 2 (HER2)-positive breast cancer, we analyzed the RNA expression profiles of HER2-positive breast cancer samples to identify the new prognostic biomarkers.

**Methods:** The linear fitting method was used to identify the differentially expressed RNAs from the HER2-positive breast cancer RNA expression profiles in the Cancer Genome Atlas (TCGA). Then, a series of methods including univariate Cox, Kaplan-Meier, and random forests, were used to identify the core long non-coding RNAs (lncRNAs) with stable prognostic value for HER2-positive breast cancer. A clinical feature analysis was performed, and a competing endogenous RNA network was constructed to explore the role of these core lncRNAs in HER2-positive breast cancer. In addition, a functional analysis of differentially expressed messenger RNAs in HER-2 positive breast cancer also provided us with some enlightening insights.

**Results:** The high expression of four core lncRNAs (AC010595.1, AC046168.1, AC069277.1, and AP000904.1) was associated with worse overall survival, while the low expression of LINC00528 and MIR762HG was associated with worse overall survival. The 6-lncRNA model has an especially good predictive power for overall survival (*p* < 0.0001) and 3-year survival (the area under the curve = 0.980) in HER2-positive breast cancer patients.

**Conclusion:** This study provides a new efficient prognostic model and biomarkers of HER2-positive breast cancer. Meanwhile, it also provides a new perspective for elucidating the molecular mechanisms underlying HER2-positive breast cancer.

## Introduction

About 15–20% of breast cancers are HER2 positive [1]. HER2 positivity, namely HER2 protein overexpression, is an important indicator of strong tumor aggressiveness and poor prognosis [2]. Due to the strong heterogeneity of breast cancer, the prognosis and treatment of HER2-positive breast cancer are significantly different from other breast cancer subgroups [3, 4]. Although new treatments have improved HER2-positive breast cancer prognosis in recent years, its persistently high mortality indicates that the situation is still alarming. Therefore, the identification of new HER2-positive breast cancer specific biomarkers is particularly urgent.

Recent comprehensive transcriptome analysis revealed that there is a specific lncRNA expression pattern in different subtypes of breast cancer [5]. The discovery of lncRNAs, ranging in length from 200 nucleotides to 100 kilobases, has helped scientists break free from their conventional view that gene regulation in biology mainly involves protein-coding genes [6, 7]. LncRNAs can regulate gene expression at the transcriptional, post-transcriptional and epigenetic levels, which in turn affects the clinical course of the disease [8]. Compared with mRNAs and microRNAs (miRNAs), lncRNAs may be a direct indicator of tumor status [9, 10]. Studies have shown that the function of lncRNAs is associated with disease progression and patient survival of cancers, such as non-small cell lung cancer, prostate cancer and gastric cancer [11–13]. Previous studies have established prognostic models for breast cancer through mRNA and alternative splicing [14, 15], but the establishment of a lncRNA system to predict HER2 positive breast cancer patients’ prognosis is hardly seen.

In this study, we applied a series of reliable bioinformatics methods to analyze the transcriptome data of HER2-positive breast cancer. Six core lncRNAs associated with stable prognosis value were identified using the randomForestSRC package and a good prognostic model was constructed. To explore the role of these core lncRNAs in HER2-positive breast cancer, we constructed a corresponding ceRNA network and performed a clinical feature analysis. In addition, we performed functional analysis of differentially expressed mRNAs and assessed the prognostic value of the core lncRNAs in various tumors in Gene Expression Profiling Interactive Analysis (GEPIA) for more comprehensive information. Features recognized in this study also helped us gain further understanding of the biological behavior of HER2-positive breast cancer at the molecular level.

## Materials and Methods

### Downloading of Human Epidermal Growth Factor Receptor 2-Positive Breast Cancer RNA Expression Data and the Corresponding Clinical Data

Highly developed microarrays and high-throughput sequencing technologies have lent powerful support for deciphering cancer genetic code [16]. TCGA provides unprecedented large-scale data for cancer researches, which provide great opportunities for revealing genomic variation in a variety of tumors [17]. RNA expression profile data of HER2-positive breast cancer samples including lncRNA, miRNA, and mRNA sequence information, were downloaded from TCGA (https://portal.gdc.cancer.gov/). These RNA data were derived from 113 HER2-positive breast cancer tissues and 105 tumor-adjacent normal breast tissues. The corresponding clinical data of the 113 HER2-positive breast cancer patients were also downloaded (Supplementary Table S1). This study has strictly followed the TCGA publication guidelines (https://www.cancer.gov/about-nci/organization/ccg/research/structural-genomics/tcga/using-tcga/citing-tcga) and all data are available without restrictions on their use in publications.

### Identification of Differentially Expressed RNAs in Human Epidermal Growth Factor Receptor 2-Positive Breast Cancer and the Functional Analysis of Differentially Expressed Messenger RNAs

We used the limma package [18] to identify differentially expressed RNAs (including lncRNAs, miRNAs, and mRNAs) between the 113 HER2-positive breast cancer tissues and 105 tumor-adjacent normal breast tissues. Then we selected the differentially expressed mRNAs for functional enrichment analysis including gene ontology (GO) analysis and Kyoto Encyclopedia of Genes and Genomes (KEGG) analysis in the R language environment [19].

### Construction and Analysis of the Protein-Protein Interaction Network

The Search Tool for the Retrieval of Interacting Genes (STRING) database (https://string-db.org) was used to construct a protein-protein interaction (PPI) network [20]. We input the names of differentially expressed mRNAs into the multiple proteins option input box of STRING database in the form of a list and selected “homo” for “species.” The interaction score of nodes was greater than 0.4. The PPI network was downloaded and visualized in Cytoscape. Furthermore, Cytoscape-MCODE was used to perform module analysis on the PPI network [21, 22]. Modules with score >4 and nodes ≥5 were selected for the functional enrichment analysis in the STRING database. Cytoscape-Centiscape was used to analyze the internal connectivity of the PPI network [23]. The statistical criterion for functional enrichment analysis was FDR <0.05.

### Identification of Long Non-Coding RNAs With Prognostic Value for Human Epidermal Growth Factor Receptor 2-Positive Breast Cancer

First, we selected lncRNAs with prognostic value for HER2-positive breast cancer patients by applying two methods, including univariate Cox regression analysis and Kaplan-Meier survival analysis (statistical criterion: *p* < 0.05). To achieve that, the survival package was used [24]. Then the overlap of the prognostic lncRNAs resulted from the above two methods was obtained. Next, the randomForestSRC package [25] was used to perform the high-dimensional variable selection for survival data on the overlapped lncRNAs, and lncRNAs with stable prognostic value for HER2-positive breast cancer were selected as core lncRNAs for subsequent analysis. Random forests is one of the classical methods for dimensionality reduction, and its results are highly reliable.

### Construction of the Prognostic Model for Human Epidermal Growth Factor Receptor 2-Positive Breast Cancer

The multiCox model was constructed using the core lncRNAs. This model was used to predict the survival risk of HER2-positive patients. The 113 HER2-positive breast cancer samples from TCGA were classified into high-risk and low-risk groups, with the median signature risk score as the cut-off value. Furthermore, we used the receiver operating characteristic (ROC) curve to evaluate the predictability of the 6-lncRNA model for the 3-year survival rate for HER2-positive breast cancer. To achieve that, the survivalROC package was used [26]. The results of the grouping, according to the classification, were compared with the actual 3-year survival status of HER2-positive breast cancer patients to obtain a false positive rate and a true positive rate. These rates were respectively represented on the abscissa (X axis) and the ordinate (Y axis) to plot the ROC curves. AUC, the area under the curve, was calculated as a measure of prognostic ability. The greater the AUC, the better the predictive ability. The risk score is the sum of the Cox regression coefficient multiplied by the expression value of each core lncRNA. The calculation formula is as follows:

$$\begin{array}{l} \;\text{Risk}\;\text{score}=\beta \;\text{lncRNA}1*\text{expression}\;\text{level}\;\text{of}\;\text{lncRNA}1+\beta \;\\ \text{lncRNA}2*\text{expression}\;\text{level}\;\text{of}\;\text{lncRNA}2+\cdots +\beta \;\text{lncRNAn}*\text{expression}\;\\ \text{level}\;\text{of}\;\text{lncRNAn}.\;\beta \;\text{is}\;\text{Cox}\;\text{regression}\;\text{coefficients}\text{.} \end{array}$$

### Construction of the Competing Endogenous RNA Network of Core Long Non-Coding RNAs Based on Weighted Gene Co-expression Network Analysis

The weighted gene co-expression network analysis (WGCNA) package [27] was used to identify the target RNAs of core lncRNAs to construct a ceRNA network [28, 29]. Cytoscape was used to implement visualization. The edge threshold was set to 0.02. Functional enrichment analysis (GO and KEGG analysis) was performed on the target mRNAs of each core lncRNA. A test result with adjusted *p* < 0.05 was considered statistically significant.

### Clinical Feature Analysis of Core Long Non-Coding RNAs and Their Prognostic Value Evaluation in Various Tumors

Currently reports on these core lncRNAs are almost non-existent. To obtain more information about the role of core lncRNAs, differences in the expression of core lncRNAs between different clinical groups were evaluated by Student’s *t* test. The data source is the 113 HER2-positive breast cancer samples originally downloaded from TCGA. These samples have differences in gender, tumor stage, lymph node metastasis, therapy, etc. An absolute value of *t* > 2 was considered to be statistically significant. Meanwhile, the prognostic value of the core lncRNAs in various tumors was assessed based on GEPIA. GEPIA (http://gepia.cancer-pku.cn) is an online server based on TCGA data. It can efficiently analyze RNA-Seq datasets of various tumor samples from TCGA [30]. A test result with logrank *p* < 0.05 was considered statistically significant.

## Results

### Differentially Expressed RNAs in Human Epidermal Growth Factor Receptor 2-Positive Breast Cancer

**FIGURE 1:**
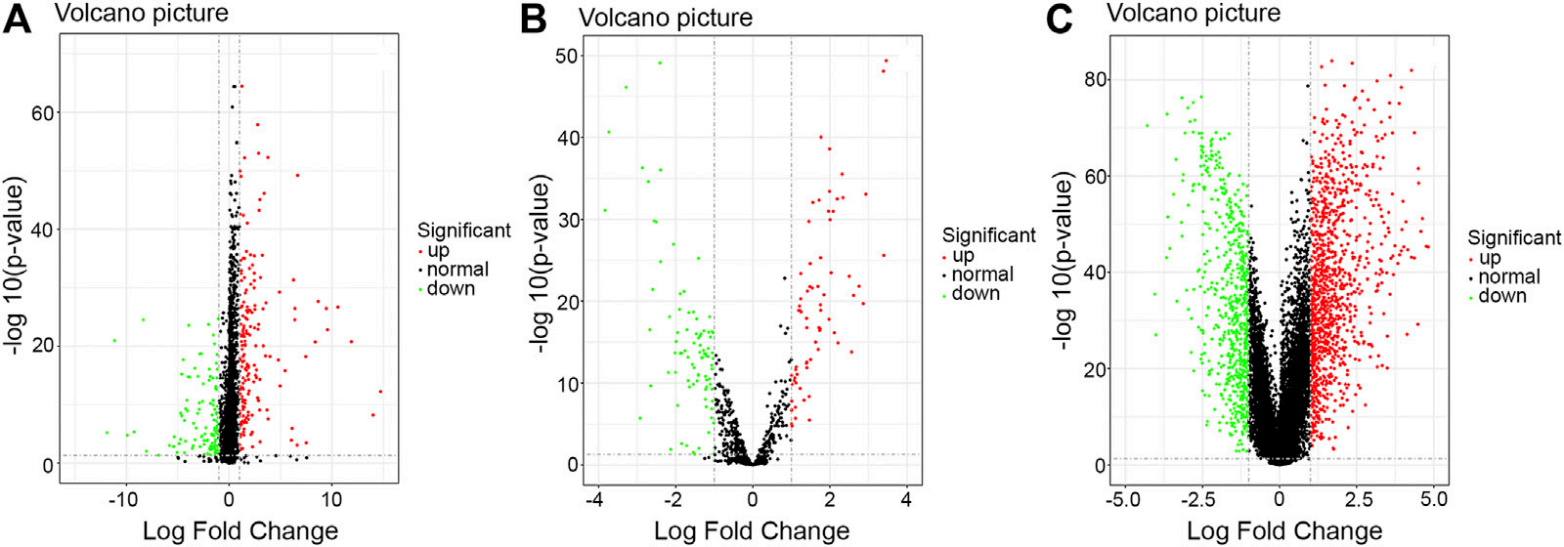
Volcano plot of the differentially expressed RNAs between HER2-positive breast cancer tissues and adjacent noncancerous breast tissues. Red: high expression; Green: low expression; Black dots: the lncRNAs with expression of |log_2_FC| < 1. *p* < 0.05. **(A)** lncRNA; (B) mi-RNA; **(C)** mRNA. Abbreviation: FC, fold change.

By using the limma package, we obtained data concerning the difference in RNA expression levels in HER2-positive breast cancer. When FDR < 0.05 and |log_2_FC| > 1 were set as the thresholds for the significance of the gene expression difference, we identified 350 differentially expressed lncRNAs, 163 differentially expressed miRNAs, and 1910 differentially expressed mRNAs in those 113 HER2-positive breast cancer samples (Figure 1).

### Results of the Functional Analysis of Differentially Expressed Messenger RNAs in Human Epidermal Growth Factor Receptor 2-Positive Breast Cancer Patients and the Analysis of the Protein-Protein Interaction Network

**FIGURE 2:**
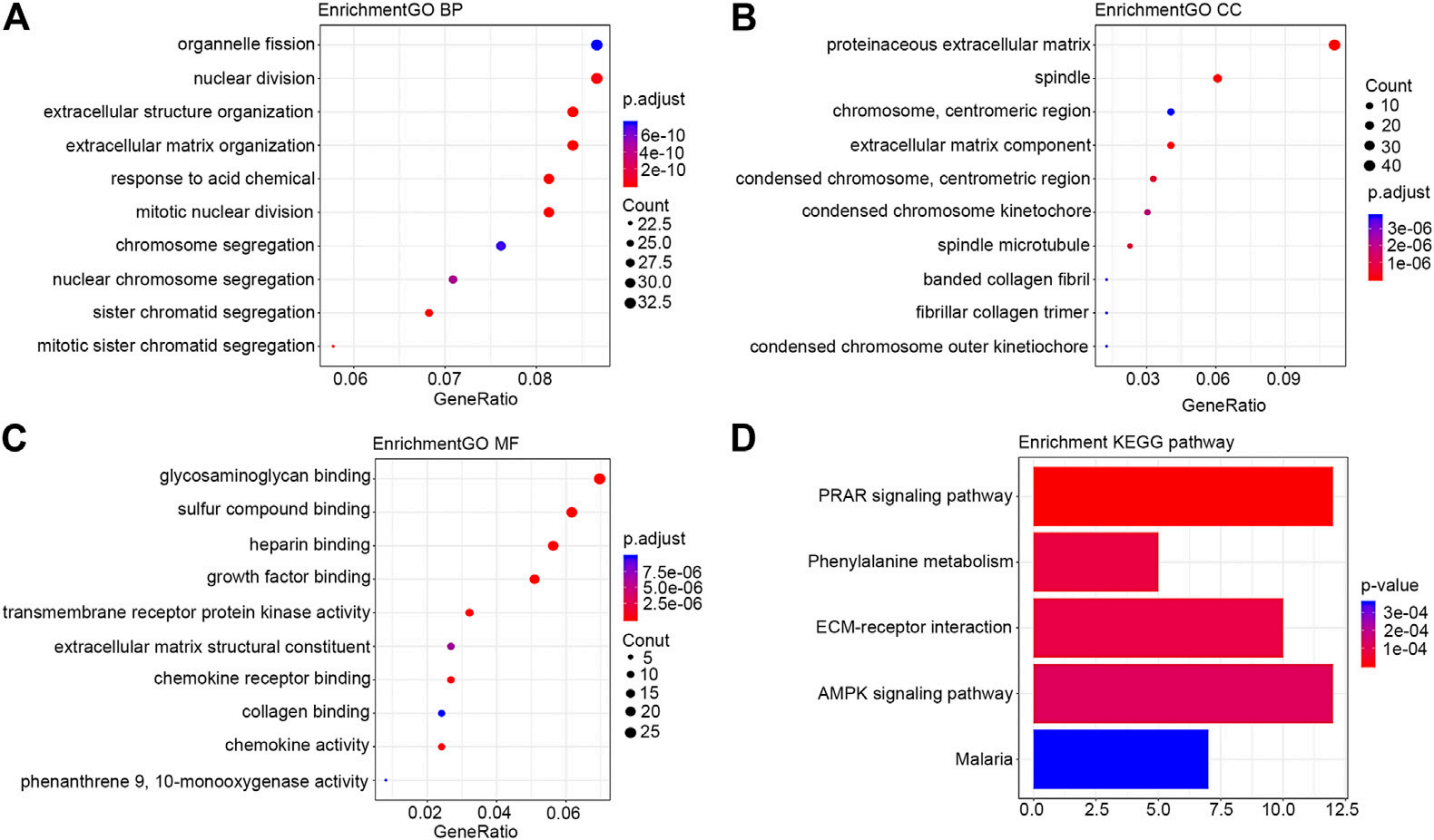
Bubble diagram of the differentially expressed mRNAs in HER2-positive breast cancer. Only results with the smallest p value are listed here. **(A)** BP; **(B)** CC; **(C)** MF; **(D)** KEGG pathway. Abbreviations: GO, gene ontology; KEGG, Kyoto Encyclopedia of Genes and Genomes; BP, biological process; MF, molecular function; CC, cell component.

The number of mRNAs selected according to the statistical criteria of FDR < 0.05 and |log_2_FC| > 1 reached 1910. In order to improve the accuracy of this study, from that pool we further selected 411 differentially expressed mRNAs based on two criteria (|log_2_FC| > 2 and FDR < 0.05) for a separate functional enrichment analysis. Results with the lowest FDR values are shown in Figure 2. The PPI network downloaded from the STRING database showed that there were significant enrichment regions (Supplementary Figure S1). Cytoscape-MCODE module analysis further showed that there were six significant modules (score >4 and nodes ≥5) in the PPI network (Supplementary Figure S2), among which the following three modules were most significant: Module 1 (score = 48.857, nodes = 50), Module 2 (score = 14, nodes = 14), and Module 3 (score = 9.357, nodes = 29). Supplementary Table S2 shows the data of the module analysis downloaded from Cytoscape.

Module 1 was primarily associated with mitosis. Module 2 was closely associated with immune functions. Module 3 was primarily involved in lipid metabolism. Module 4 was primarily involved in cell differentiation, stratum corneum formation, and signaling by Receptor Tyrosine Kinases. Module 5 was involved in post-translational protein phosphorylation. Module 6 participated in digestion and absorption, and degradation of the extracellular matrix. The results of the modules’ functional analysis are included in Supplementary Table S3. The connectivity analysis by Cytoscape-Centiscape of the PPI network showed that 88 differentially expressed mRNAs had a degree greater than 20, and they mainly concentrated in Module 1 (50), Module 2 (5), Module 3 (15), Module 4 (10), and Module 6 (5). The main results of the connectivity analysis are included in Supplementary Table S4.

### The Core Long Non-Coding RNAs With Stable Prognostic Value for Human Epidermal Growth Factor Receptor 2-Positive Breast Cancer

**FIGURE 3:**
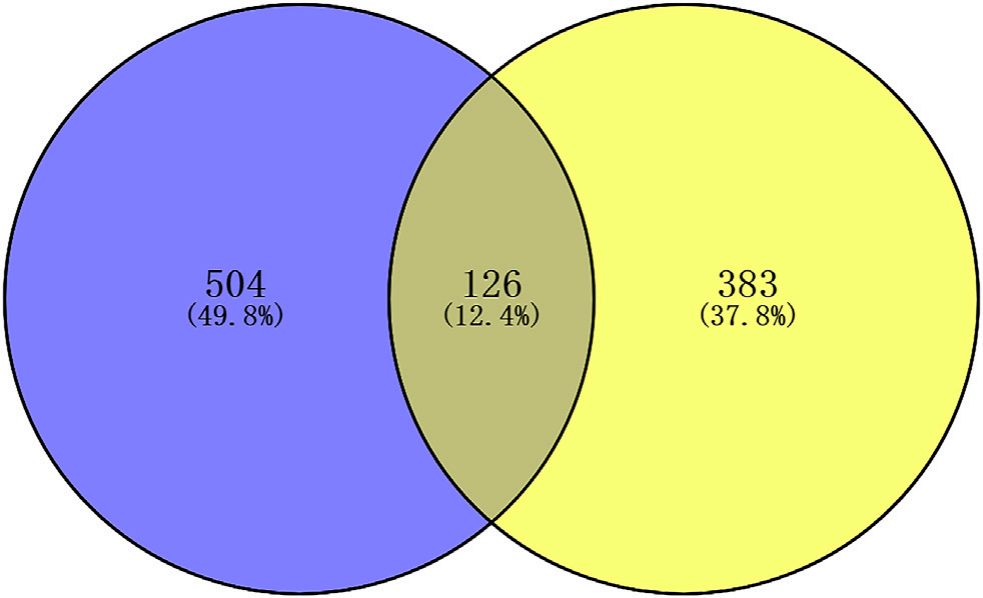
Venn plot of the prognostic lncRNAs identified by two methods. Blue: univariate Cox regression analysis; yellow: Kaplan-Meier survival analysis. *p* < 0.05.

There were 504 lncRNAs with significant prognostic value for HER2-positive breast cancer by the univariate Cox regression analysis. Results from 383 lncRNAs indicated significant prognostic value for HER2-positive breast cancer by Kaplan-Meier survival analysis. The number of the overlap of the lncRNAs identified by the two methods was 126. Figure 3 is plotted with the Venn online software (https://bioinfogp.cnb.csic.es/tools/venny/index.html). After a high-dimensional selection of the survival random variables was performed on the 126 lncRNAs using the randomForestSRC package, and the following six stable survival-related lncRNAs were obtained: AC010595.1, AC046168.1, AC069277.1, AP000904.1, LINC00528, and MIR762HG. In consideration of the differential expression analysis, we found that the expression levels of five core lncRNAs (AC010595.1, AC046168.1, AC069277.1, AP000904.1 and MIR762HG) in HER2-positive breast cancer tissues were higher than those in adjacent normal breast tissues (|t| > 2 and FDR <0.05), while there was no significant difference in the expression of LINC00528. Taking into account the results of clinical feature analysis, this may be because the expression level of LINC00528 in early HER2-positive breast cancer (T1/2) was higher than advanced HER2-positive breast cancer (T3/4) (|t| = 1.930803441, *p* = 0.060616568). The details of these six core lncRNAs are shown in Tables 1 and 2.

**TABLE 1:** The survival analysis results of the core lncRNAs.

Information	Cox analysis		Survival different		RSF-VH analysis		
Name	Coefficient	*p* values	Chisq values	*p* values	Depth	Rel. freq	VI
AC010595.1	4.39	0.00032	3.95	0.047	4.868	16	0.01312666
AC046168.1	5.56	0.0000052	5.57	0.018	4.627	18	0.04929882
AC069277.1	20.61	0.00019	6.18	0.013	5.042	18	0.03784634
AP000904.1	13.89	3.99E-06	7.58	0.0059	3.921	17	0.03686783
LINC00528	−18.45	0.027	5.44	0.019	5.308	17	0.03090886
MIR762HG	−4.09	0.024	4.05	0.044	5.206	29	0.04322642

Chisq, hi-square; RSF-VH, random survival forests-variable hunting, Rel. freq, Relative frequency; VI, variable importance.

**TABLE 2:** The expression of core lncRNAs in HER2-positive breast cancer.

Symbol	Log_2_FC	*t*	*p* value	FDR
AC010595.1	−0.076688216	−4.556493508	8.69E-06	3.85E-05
AC046168.1	−0.034387366	−2.335474983	0.020433454	0.045385091
AC069277.1	−0.007222874	−2.40156115	0.017169908	0.038882814
AP000904.1	−0.029456815	−4.435858207	1.46E-05	6.23E-05
LINC00528	0.009987881	0.948836716	0.343761433	0.458696976
MIR762HG	−0.154237904	−3.930359618	0.000114125	0.000419305

FC, fold change; FDR, false discovery rate.

### The Prognostic Model of Human Epidermal Growth Factor Receptor 2-Positive Breast Cancer

**FIGURE 4:**
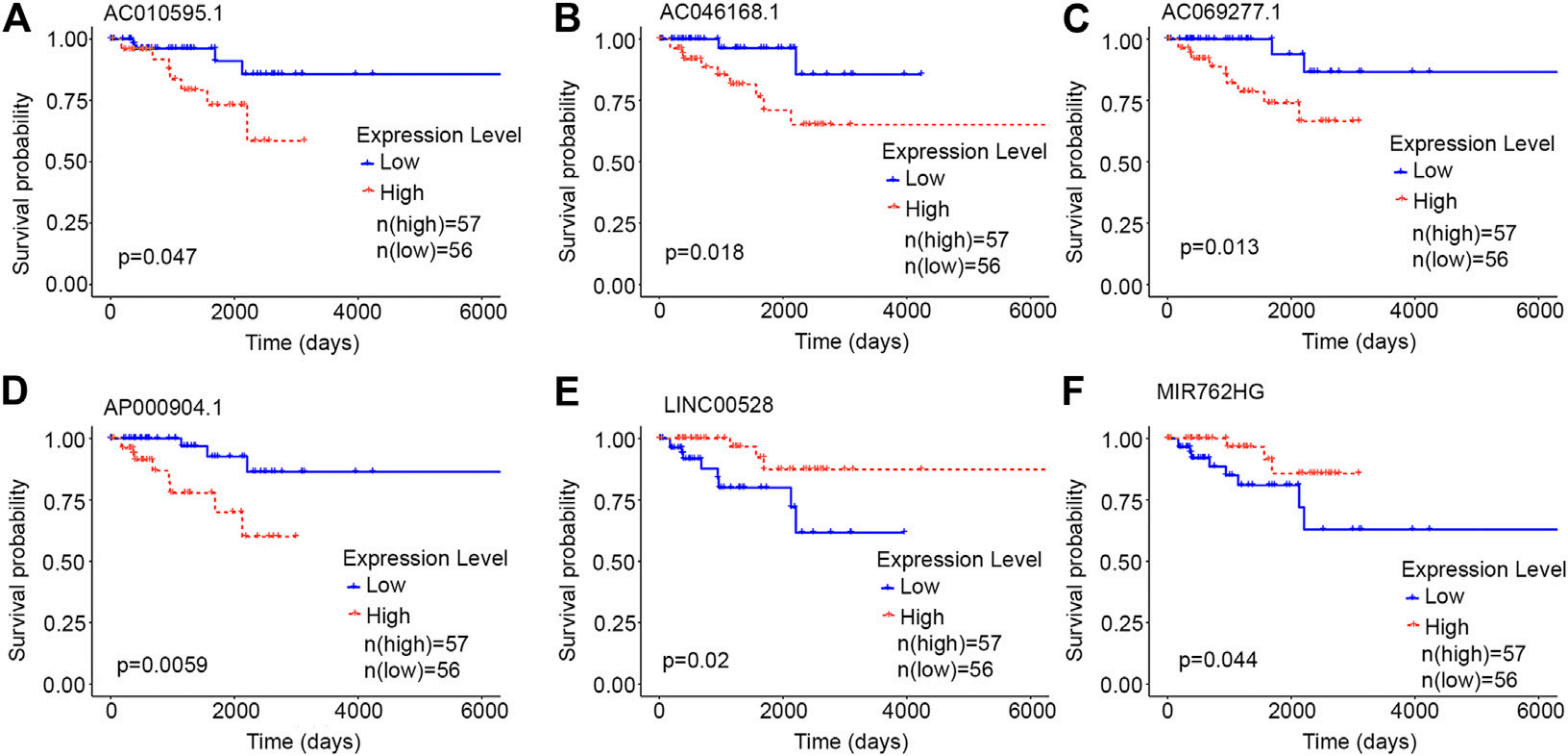
Kaplan-Meier curves of the core lncRNAs in HER2-positive breast cancer. The Kaplan-Meier curves show the prognostic significance of the core lncRNAs in patients with HER2-positive breast cancer. The red lines represent patients with a high gene expression, and blue lines represent patients with a low gene expression. **(A)** AC010595.1; **(B)** AC046168.1; **(C)** AC069277.1; **(D)** AP000904.1; **(E)** LINC00528; **(F)** MIR762HG.

**FIGURE 5:**
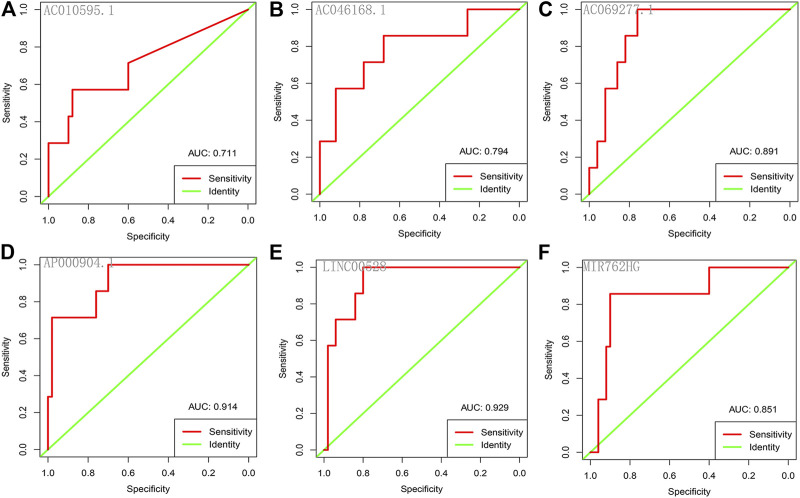
ROC curves of the core lncRNAs in HER2-positive breast cancer. The ROC curves show the core lncRNAs’ prognostic ability for 3-year survival in patients with HER2-positive breast cancer. Red: sensitivity. Green: identity. X axis (specificity): false positive rate. Y axis (sensitivity): true positive rate. AUC: area under curve. **(A)** AC010595.1; **(B)** AC046168.1; **(C)** AC069277.1; **(D)** AP000904.1; **(E)** LINC00528; **(F)** MIR762HG.

Each of the six core lncRNAs had a good single-factor prognosis for overall survival and 3-year survival in HER2-positive breast cancer patients (Figure 4: *p* < 0.05; Figure 5: AUC > 0.7). The multiCox model of the six core lncRNAs (AC010595.1, AC046168.1, AC069277.1, AP000904.1, LINC00528, and MIR762HG) had a high-level capability of risk assessment for HER2-positive breast cancer patients. The formula for determining the risk score is given below:

$$\begin{array}{l} \;\text{Risk}\;\text{score}=(4.39*\text{expression}\;\text{level}\;\text{of}\;\text{AC}010595\text{.}1)\\ +(5.56*\text{expression}\;\text{level}\;\text{of}\text{AC}046168\text{.}1)\\ +(20.61*\text{expression}\;\text{level}\;\text{of}\;\text{AC}069277.1)\\ +(13.89*\text{expression}\;\text{level}\;\text{of}\text{AP}000904.1)\\ +(-18.45*\text{expression}\;\text{level}\;\text{of}\;\text{LINC}00528)\\ +(-4.09*\text{expression}\;\text{level}\;\text{of}\text{MIR}762\text{HG}). \end{array}$$

**FIGURE 6:**
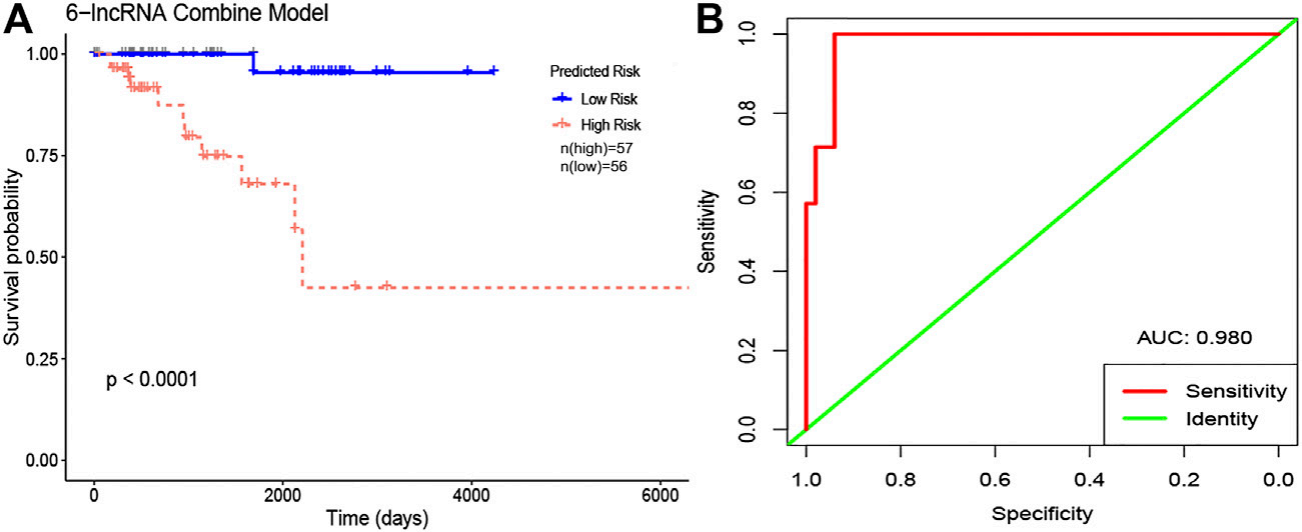
The Kaplan-Meier curve and ROC curve of 6-lncRNA combine model in HER2-positive breast cancer. **(A)** Kaplan-Meier curves. The red lines represent patients with high risk, and blue lines represent patients with low risk. **(B)** ROC curves. Red: sensitivity. Green: identity. X axis (specificity): false positive rate. Y axis (sensitivity): true positive rate. Abbreviation: AUC, area under curve.

According to the median signature risk score, the 113 HER2-positive breast cancer samples from TCGA were divided into high-risk and low-risk groups with significant overall survival differences (*p* < 0.0001), and the ROC curve showed that the 3-year survival rates were predicted with high accuracy (AUC = 0.980) (Figure 6).

### The Competing Endogenous RNA Network of Core Long Non-Coding RNAs and Results of the Functional Enrichment Analysis

**FIGURE 7:**
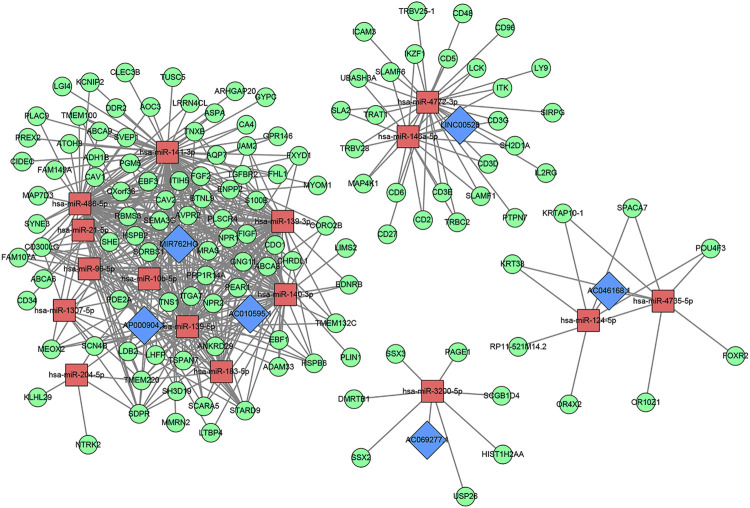
The core lncRNA-miRNA-mRNA network based on ceRNA mechanism. Blue: lncRNA; Red: miRNA; Green: mRNA.

Sixteen miRNAs and 129 mRNAs were identified for the construction of the ceRNA network of core lncRNAs. Figure 7 shows the ceRNA network. Three core lncRNAs (AC010595.1, AP000904.1 and MIR762HG) were closely associated in the ceRNA network, and the functional analysis results of their target mRNAs were similar: they were involved in biological processes including muscle organ development, regulation of vasculature development, and angiogenesis. They were enriched in KEGG pathways of proteoglycans in cancer, cGMP-PKG signaling pathway. They were also closely associated with focal adhesion. Notably, the KEGG analysis showed that target mRNAs of AP000904.1 were also enriched in the PI3K-Akt signaling pathway and Ras signaling pathway. Moreover, FGF2 (|log_2_FC| = 2.60345034144707, FDR = 7.58730025761752E-75), the famous cancer-related gene, was found to be a target mRNA of three core lncRNAs (AC010595.1, AP000904.1 and MIR762HG) in the ceRNA network. Based on the above analysis, we also discovered that FGF2 had a high connectivity in the PPI network and was subordinate to Module 3 (degree = 58).

The GO analysis showed that the target mRNAs of LINC00528 were involved in biology processes activated by immune cells such as T cells, lymphocytes and leukocytes, and were enriched in positive regulation of leukocyte cell-cell adhesion. The KEGG analysis showed that these target mRNAs were also enriched in hematopoietic cell lineage, besides being involved in primary immunodeficiency, Th1 and Th2 cell differentiation, and T cell receptor signaling pathway.

The functional analysis showed that the target mRNAs of AC046168.1 participated in the biology processes of epidermal cell differentiation, and epidermis development. The target mRNAs of AC069277.1 were associated with epigenetics, and were involved in the biology process of transcription corepressor activity and KEGG pathways of transcriptional mis-regulation in cancer, and necroptosis. Table 3 lists the primary results of the functional enrichment analysis of target mRNAs of each core lncRNA. A Q-value less than 0.05 is considered statistically significant. Supplementary Figures S3–S8 show the bubble diagrams.

**TABLE 3:** The main functional analysis results of the target mRNAs of each core lncRNA.

ID	Description	Q value	Count
AC010595.1			
Biology process			
GO:0007517	Muscle organ development	0.003517703	8
GO:1901342	Regulation of vasculature development	0.003517703	7
GO:0001525	Angiogenesis	0.003517703	8
GO:0061337	Cardiac conduction	0.003736857	5
Celluar component			
GO:0005901	Caveola	0.002572495	4
GO:0044853	Plasma membrane raft	0.002572495	4
GO:0045121	Membrane raft	0.002572495	6
GO:0098857	Membrane microdomain	0.002572495	6
GO:0098589	Membrane region	0.005744063	6
GO:0001653	Peptide receptor activity	0.014043265	4
KEGG pathway			
hsa05205	Proteoglycans in cancer	0.021546616	5
hsa04022	cGMP-PKG signaling pathway	0.05294302	4
hsa04510	Focal adhesion	0.068542716	4
AC046168.1			
Biology process			
GO:0009913	Epidermal cell differentiation	0.006405587	3
GO:0008544	Epidermis development	0.006980753	3
AP000904.1			
Biology process			
GO:0007517	Muscle organ development	0.004405768	8
GO:1901342	Regulation of vasculature development	0.004405768	7
GO:0001525	Angiogenesis	0.004405768	8
Cellular component			
GO:0005901	Caveola	0.003856935	4
GO:0044853	Plasma membrane raft	0.003856935	4
GO:0045121	Membrane raft	0.003856935	6
GO:0098857	Membrane microdomain	0.003856935	6
GO:0098589	Membrane region	0.008552471	6
GO:0043235	Receptor complex	0.019729483	5
GO:0001653	Peptide receptor activity	0.017068444	4
KEGG pathway			
hsa05205	Proteoglycans in cancer	0.031738163	5
hsa04022	cGMP-PKG signaling pathway	0.071853414	4
hsa04510	Focal adhesion	0.081765703	4
hsa04151	PI3K-Akt signaling pathway	0.081765703	5
hsa04014	Ras signaling pathway	0.081765703	4
LINC00528			
Biology process			
GO:0042110	T cell activation	7.85E-14	14
GO:0051249	Regulation of lymphocyte activation	4.69E-11	12
GO:0002696	Positive regulation of leukocyte activation	5.43E-11	11
GO:0050867	Positive regulation of cell activation	5.90E-11	11
GO:0051251	Positive regulation of lymphocyte activation	4.14E-10	10
GO:0050870	Positive regulation of T cell activation	4.14E-10	9
GO:0050863	Regulation of T cell activation	4.14E-10	10
GO:0030098	Lymphocyte differentiation	4.35E-10	10
GO:1903039	Positive regulation of leukocyte cell-cell adhesion	4.70E-10	9
GO:1903037	Regulation of leukocyte cell-cell adhesion	5.41E-10	10
Cellular component			
GO:0098552	Side of membrane	2.59E-10	11
GO:0042101	T cell receptor complex	4.08E-10	5
GO:0009897	External side of plasma membrane	3.02E-07	7
GO:0009898	Cytoplasmic side of plasma membrane	1.37E-05	5
GO:0098802	Plasma membrane receptor complex	1.39E-05	5
GO:0098562	Cytoplasmic side of membrane	1.64E-05	5
GO:0043235	Receptor complex	0.000173207	5
KEGG pathway			
hsa05162	Measles	2.59E-06	6
hsa05340	Primary immunodeficiency	4.06E-06	4
hsa04658	Th1 and Th2 cell differentiation	4.06E-06	5
hsa04640	Hematopoietic cell lineage	4.06E-06	5
hsa04660	T cell receptor signaling pathway	4.06E-06	5
MIR762HG			
Biology process			
GO:0007517	Muscle organ development	0.004744758	10
GO:1901342	Regulation of vasculature development	0.004744758	9
GO:0001667	Ameboidal-type cell migration	0.007904947	9
GO:0045765	Regulation of angiogenesis	0.007904947	8
GO:0001525	Angiogenesis	0.008004132	10
GO:0061337	Cardiac conduction	0.008004132	6
Cellular component			
GO:0005925	Focal adhesion	0.010229707	8
GO:0005924	Cell-substrate adherens junction	0.010229707	8
GO:0005901	Caveola	0.010229707	4
GO:0030055	Cell-substrate junction	0.010229707	8
GO:0044853	Plasma membrane raft	0.019787068	4
GO:0045121	Membrane raft	0.030004811	6
GO:0098857	Membrane microdomain	0.030004811	6
GO:0005911	Cell-cell junction	0.030624509	7
GO:0044325	Ion channel binding	0.010135833	5
GO:0005539	Glycosaminoglycan binding	0.039860382	5
GO:0001653	Peptide receptor activity	0.039860382	4
GO:0016247	Channel regulator activity	0.039860382	4
KEGG pathway			
hsa05205	Proteoglycans in cancer	0.066608026	5
hsa04022	cGMP-PKG signaling pathway	0.128309315	4

KEGG, Kyoto Encyclopedia of Genes and Genomes.

### Results of the Clinical Feature Analysis of Core Long Non-Coding RNAs and Their Prognostic Value in Various Tumors Based on Gene Expression Profiling Interactive Analysis

The results from the Student’s *t*-test showed that the six core lncRNAs were associated with some clinical parameters of HER2-positive breast cancer. It is worth noting that the expression level of LINC00528, to a certain extent, can distinguish between patients with early HER2-positive breast cancer and patients with advanced HER2-positive breast cancer: the expression level of LINC00528 in early HER2-positive breast cancer patients (T1/2) was higher than that in advanced HER2-positive breast cancer patients (T3/4) (t = 1.930803441, *p* = 0.060616568). Compared with non-targeted molecular therapy group, the expression level of LINC00528 was significantly higher in the targeted molecular therapy group (|t| > 2). These results are consistent with the better prognosis in the high expression group of LINC00528. The details are shown in Supplementary Table S5.

**FIGURE 8:**
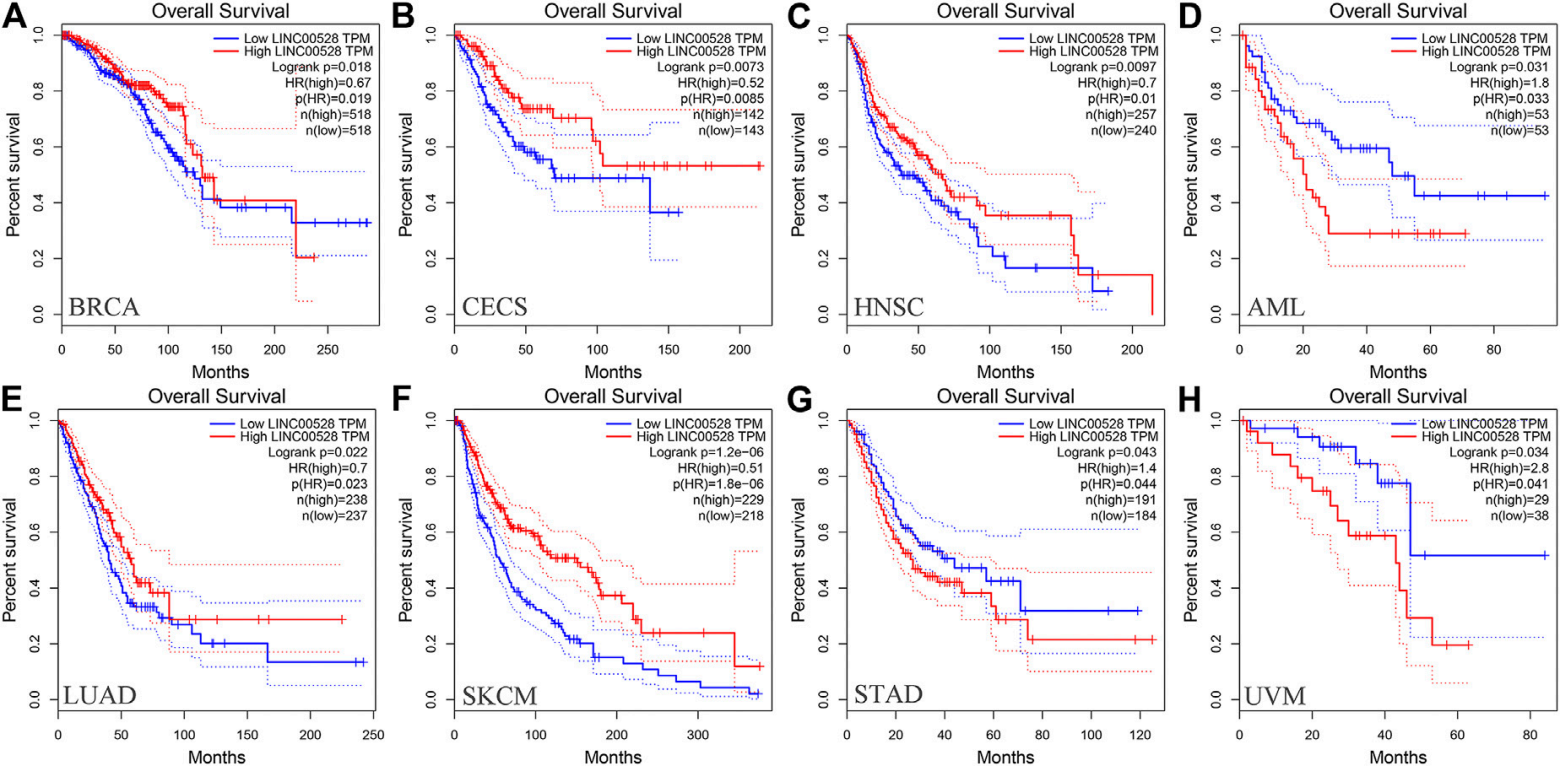
Kaplan-Meier curves of the core lncRNA LINC00528 in various cancers based on GEPIA. The Kaplan-Meier curves show the prognostic significance of LINC00528 in a variety of cancers according to the survival analysis based on GEPIA. The red lines represent patients with high gene expression, and blue lines represent patients with a low gene expression. **(A)** BRCA: breast invasive carcinoma; **(B)** CECS: cervical squamous cell carcinoma and endocervical adenocarcinoma; **(C)** HNSC: head and neck squamous cell carcinoma; **(D)** AML: acute myeloid leukemia; **(E)** LUAD: lung adenocarcinoma; **(F)** SKCM: skin cutaneous melanoma; **(G)** STAD: stomach adenocarcinoma; **(H)** UVM: uveal melanoma. Abbreviations: GEPIA, Gene Expression Profiling Interactive Analysis.

At present in GEPIA (http://gepia.cancer-pku.cn/), only relevant information of LINC00528 was available while the data of the other five core lncRNAs were not. We analyzed the prognostic value of LINC00528 for all 31 diseases included by GEPIA and found that the high expression of LINC00528 was associated with better overall survival in breast invasive carcinoma, cervical squamous cell carcinoma and endocervical adenocarcinoma, head and neck squamous cell carcinoma, lung adenocarcinoma and skin cutaneous melanoma, while the high expression of LINC00528 was associated with worse overall survival in acute myeloid leukemia, stomach adenocarcinoma and uveal melanoma (logrank *p* < 0.05). These results were downloaded from GEPIA. Figure 8 shows the details. The references for analysis platforms and packages were provided in Table 4.

**TABLE 4:** The summary table with references for analysis platforms and packages used in the methodology.

Platform or package	References
TCGA	https://portal.gdc.cancer.gov/
R	[19]
Limma package	[18]
Search tool for the retrieval of interacting genes	https://string-db.org/cgi/input?sessionId=bgfaqcKYCOJ4&input_page_show_search=on
Cytoscape-MCODE	[22]
Cytoscape-centiscape	[23]
RandomForestSRC package	[25]
GEPIA (gepia.cancer-pku.cn)	[30]
Venn	https://bioinfogp.cnb.csic.es/tools/venny/index.html
WGCNA package (https://CRAN.R-project.org/package=WGCNA)	[27]
Survival package (https://CRAN.R-project.org/package=survival)	[24]
Survival ROC package (https://CRAN.R-project.org/package=survivalROC)	[26]

## Discussion

The continuous advancement of molecular oncology has made it possible to identify the genetic characteristics of tumor subtypes. These genetic characteristics are often closely associated with the activity and therapeutic opportunities of oncogenic pathways, hence becoming important biomarkers and drug targets for tumors [31]. In combination with the high heterogeneity of breast cancer genomes, the identification of subtype-specific related gene markers can provide important support for clinical strategies to improve breast cancer subtypes. Currently, advanced bioinformatics tools based on sophisticated algorithms are constantly available, allowing us to identify potential driving events in diseases from massive genomic data [32–34]. Based on transcriptome data, this study identified key biomarkers closely related to the prognosis of HER2-positive breast cancer and explored the molecular pathogenesis of HER2-positive breast cancer.

The potential of lncRNA as a prognostic factor for tumors has been recognized. As a complement to mRNAs and miRNAs, lncRNA-rich transcripts provide us with important materials for obtaining tumor prognostic factors. Through reliable analysis methods such as univariate Cox, Kaplan-Meier and random forests, we eventually identified the following six stable survival-related lncRNAs as core lncRNAs in HER2-positive breast cancer: AC010595.1, AC046168.1, AC069277.1, AP000904.1, LINC00528, and MIR762HG. We associated the high expression of four core lncRNAs (AC010595.1, AC046168.1, AC069277.1, and AP000904.1) with worse overall survival, from which we inferred that they promoted the development of cancer. The low expression of LINC00528 and MIR762HG was associated with worse overall survival, suggesting that they may have been tumor suppressor genes. Each of the core lncRNAs had good predictive power for 3-year survival, with AP000904.1 (AUC = 0.914) and LINC00528 (AUC = 0.929) being optimal.

The multi-factor regression model composed of these six core lncRNAs has a non-negligible advantage. It has a very good risk assessment ability of HER2-positive breast cancer patients (*p* < 0.05), and a powerful prognostic ability for 3-year survival (AUC = 0.980). This provides an effective classification tool for the clinical prognosis assessment of HER2-positive breast cancer. We also intended to test the prognostic model using an independent data set in Gene Expression Omnibus. Unfortunately, we were not able to find a fully applicable data set. This is partly because there are few related studies on these six core lncRNAs. The lack of large clinical cohorts to validate the model is one of the limitations of this study. However, considering that the six core lncRNAs in this study have shown stable prognostic value for HER2-positive breast cancer in a variety of prognostic analyses, the 6-lncRNA prognostic model is relatively reliable and worthy of further study.

Currently, non-coding regions are not fully annotated and we could hardly find any reports on these six prognostic lncRNAs. Therefore, our understanding of these core lncRNAs is not sufficiently comprehensive. More researches and tests are needed for clinical transformation. Non-coding genes usually function in complex regulatory networks [35]. To explore the functional relevance of core lncRNAs in HER2-positive breast cancer, we constructed a corresponding ceRNA network. Three core lncRNAs (AC010595.1, AP000904.1, and MIR762HG) were found closely related in the ceRNA network. Their target mRNAs were significantly enriched in the biological processes of muscle organ development, regulation of vasculature development, and angiogenesis, and closely associated with focal adhesion. In consideration of the importance of angiogenesis and cell adhesion in the pathogenesis of tumors, it could be inferred that AC010595.1, AP000904.1 and MIR762HG have jointly affected the growth and metastasis of HER2-positive breast cancer through ceRNA mechanisms. Furthermore, the famous cancer-related gene FGF2 was found to be a target mRNA of the three core lncRNAs in the ceRNA network. FGF2, an important member of the tyrosine kinase receptor family, plays a key role in the development of tumors. By reviewing the other analysis results in this study, we also identified that FGF2 was a low expressed mRNA in HER2-positive breast cancer (|log_2_FC| = 2.60345034144707, FDR = 7.58730025761752E-75) and occupied a core position in the PPI network (degree = 58). These newly discovered relationships between FGF2 and the core lncRNAs are valuable.

In addition, the KEGG analysis also showed AP000904.1 target mRNAs directly participated in the PI3K-Akt signaling pathway as well as the Ras signaling pathway. It is well known that these two pathways are the classical signal transduction pathways activated mainly by HER-2 and other members of the HER/ErBb receptor family after they form heterodimers. They can form cross-talk with other signaling pathways, and participate in biological functions including cell proliferation, migration, infiltration, anti-apoptosis and promotion of angiogenesis [36]. AP000904.1 is likely to be a key regulator for HER2 overexpressing tumors that deserves more attention.

The functional enrichment analysis showed that target mRNAs of LINC00528 were closely associated with immune activity and they participated in biology processes activated by various immune cells such as T cells, lymphocytes and leukocytes. LINC00528 was especially enriched in the biology processes of positive regulation of leukocyte cell-cell adhesion and regulation of leukocyte cell-cell adhesion (including multiple molecules of the CD family). This suggests that LINC00528 is closely associated with tumor cell metastasis. Based on the results of prognostic analysis and clinical characterization, we hypothesized that the low expression level of LINC00528 at the late stage is likely to be an important factor for the late metastasis of HER2-positive breast cancer. Survival analysis based on GEPIA also showed that LINC00528 had a good prognostic value in various tumors (*p* < 0.05), which suggested that LINC00528 could play an important role in the mechanism of cancer metastasis.

Functional analysis of differentially expressed mRNAs has also provided us with some enlightening insights. Several differentially expressed mRNAs in HER2-positive breast cancer (including CXCL2, CXCL12, CXCL10, CXCL11, CXCL9 and CXCL14) were enriched in the biology processes of chemokine receptor binding and chemokine activity. The result provides a molecular basis for elucidating the metastatic pattern of HER2-positive breast cancer. Interestingly, the differentially expressed mRNAs were significantly enriched in the malaria KEGG pathway (FDR = 0.0149807601977498). Previous studies have shown that anti-malarial drugs can cause cell cycle arrest and apoptosis in HER2-enriched breast cancer cells [37]. Furthermore, the PPI network showed modules which were significant for the exploration of the biological behavior of HER2-positive breast cancer. Module 1 was the most significant and primarily involved in mitosis, which is consistent with the characteristics of HER2 overexpression. Module 2 showed the immune regulation in HER2-positive breast cancer. Notably, Module 3 participated in the Adenosine 5′-monophosphate-activated protein kinase (AMPK) signaling pathway. AMPK as a regulator of cellular energy metabolism is an ideal target for tumor therapy [38, 39].

## Conclusion

This study provides a new efficient prognostic model of HER2-positive breast cancer. The 6-lncRNA prognostic model is relatively reliable and worthy of further study. The six core lncRNAs are also potential drug targets. Currently there are almost no reports regarding the six core lncRNAs. This study has thus laid a foundation for a further understanding of the roles of these core lncRNAs and for obtaining the key pathways. Meanwhile, it provides a new perspective for elucidating the molecular mechanisms underlying HER2-positive breast cancer.

## Data Availability

Publicly available datasets were analyzed in this study. This data can be found here: https://portal.gdc.cancer.gov/. Code availability: We conducted data analysis in R (3.4.0) language environment. The R packages used are available on the website: https://mirrors.tuna.tsinghua.edu.cn/CRAN/. We also applied GEPIA (http://gepia.cancer-pku.cn/) for further analysis, which has been explained appropriately in the paper.

## Abbreviations

HER2, human epidermal growth factor receptor 2; TCGA, the Cancer Genome Atlas; lncRNA, long non-coding RNA; ceRNA, competing endogenous RNA; mRNA, messenger RNA; ROC, receiver operating characteristic; AUC, area under the curve; miRNA, microRNA; GEPIA, Gene Expression Profiling Interactive Analysis; GO, gene ontology; KEGG, Kyoto Encyclopedia of Genes and Genomes; STRING, search tool for the retrieval of interacting genes; PPI, protein-protein interaction; WGCNA, weighted gene co-expression network analysis; AMPK, Adenosine 5′-monophosphate (AMP)-activated protein kinase
