# Supplementary material for: 6-lncRNA Assessment Model for Monitoring and Prognosis of HER2-Positive Breast Cancer: Based on Transcriptome Data
Source: Pathol Oncol Res. 2021 Apr 13;27:609083. doi: 10.3389/pore.2021.609083 (PMC8262145; doi:10.3389/pore.2021.609083)
Supplement: Supplementary file 1 [file DataSheet1.ZIP › Supplementary materials/Fig S1.pdf]

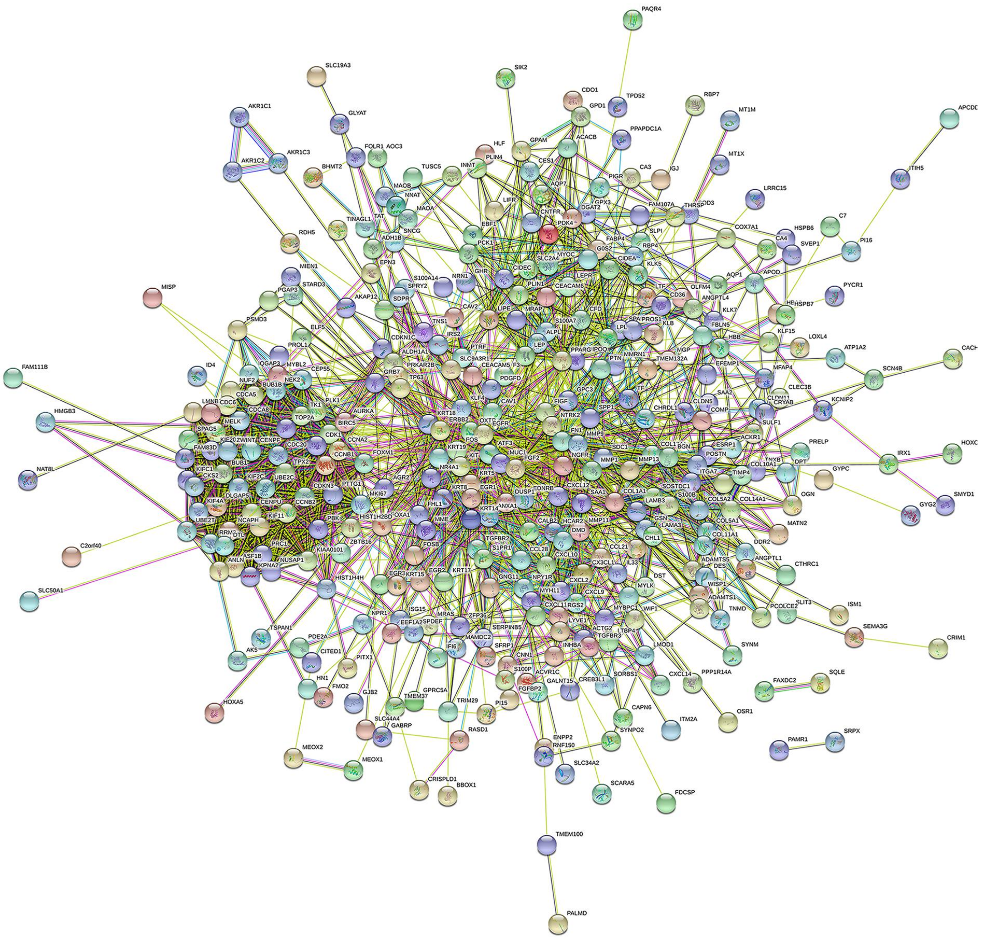

Figure S1 The PPI network of differentially expressed mRNAs in HER2-positive breast cancer. Interaction score > 0.4. **Abbreviation**: PPI, protein-protein interaction.
