# Supplementary material for: 6-lncRNA Assessment Model for Monitoring and Prognosis of HER2-Positive Breast Cancer: Based on Transcriptome Data
Source: Pathol Oncol Res. 2021 Apr 13;27:609083. doi: 10.3389/pore.2021.609083 (PMC8262145; doi:10.3389/pore.2021.609083)
Supplement: Supplementary file 1 [file DataSheet1.ZIP › Supplementary materials/Fig S3.pdf]

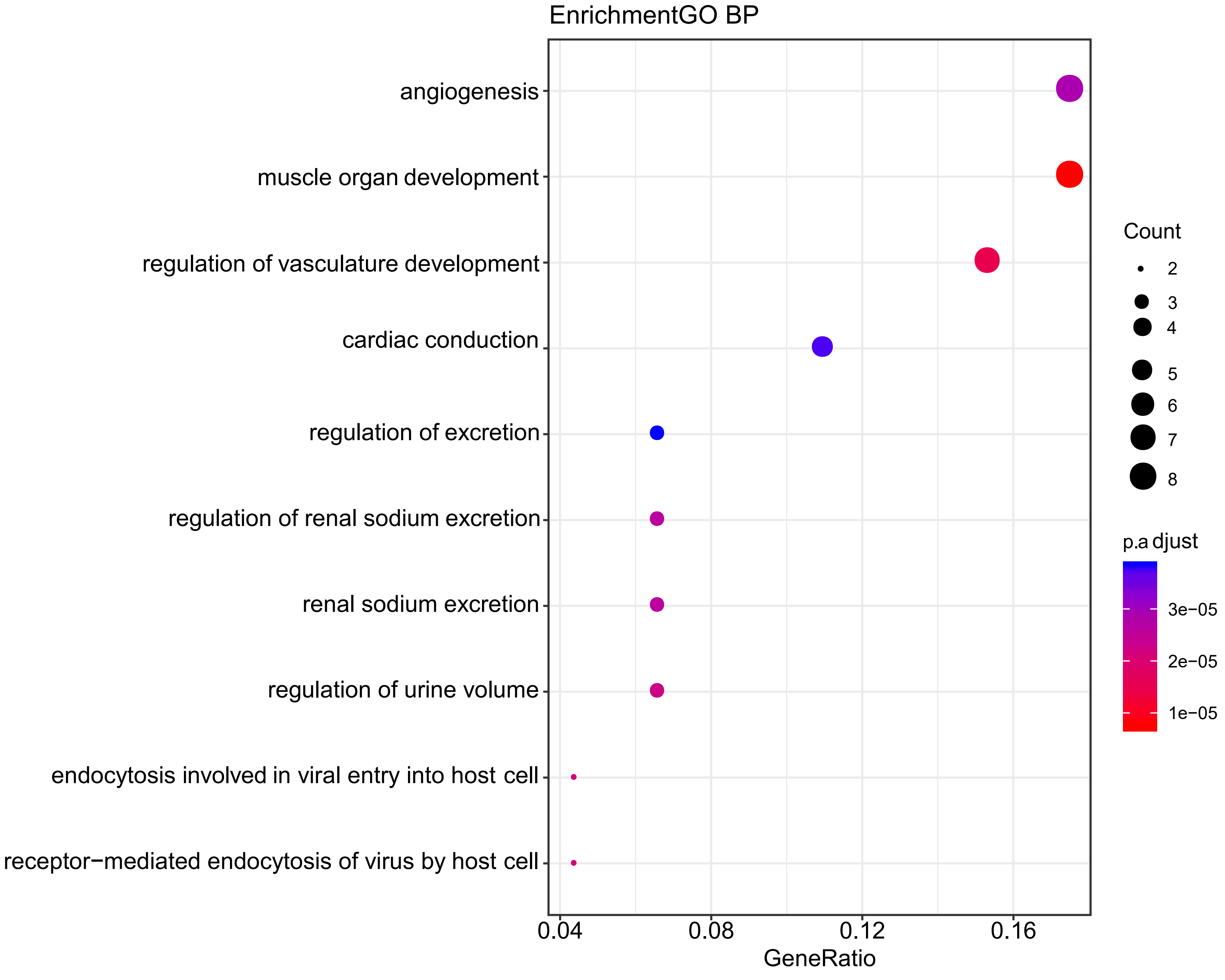

EnrichmentGO BP
angiogenesis
muscle organ development
regulation of vasculature development
cardiac conduction
regulation of excretion
regulation of renal sodium excretion
renal sodium excretion
regulation of urine volume
endocytosis involved in viral entry into host cell
receptor−mediated endocytosis of virus by host cell
0.04
0.08
0.12
0.16
GeneRatio
Count
2
3
4
5
6
7
8
p.adjust
3e−05
2e−05
1e−05

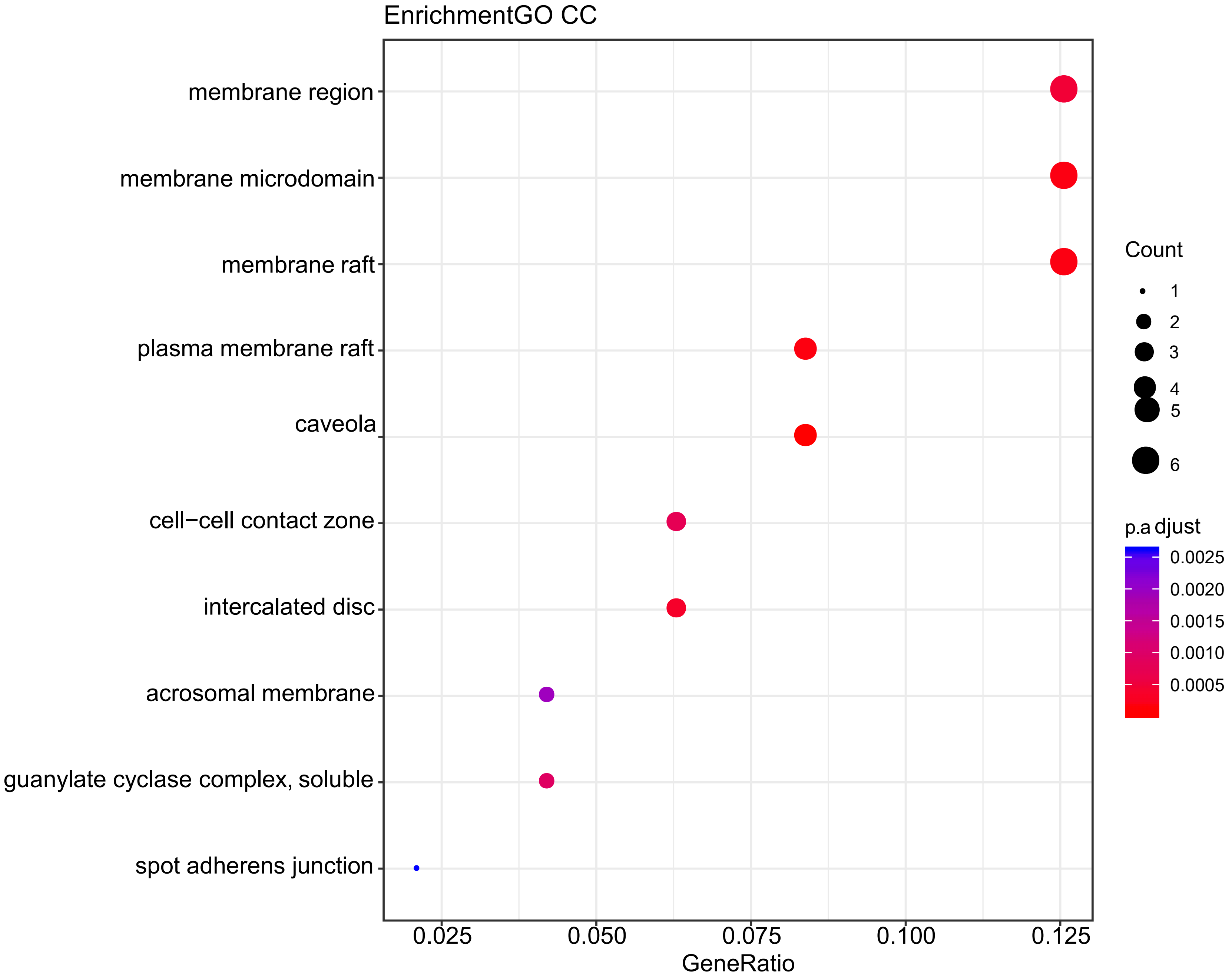
EnrichmentGO CC
membrane region
membrane microdomain
membrane raft
plasma membrane raft
caveola
cell−cell contact zone
intercalated disc
acrosomal membrane
guanylate cyclase complex, soluble
spot adherens junction
0.025
0.050
0.075
0.100
0.125
GeneRatio
Count
1
2
3
4
5
6
p.adjust
0.0025
0.0020
0.0015
0.0010
0.0005

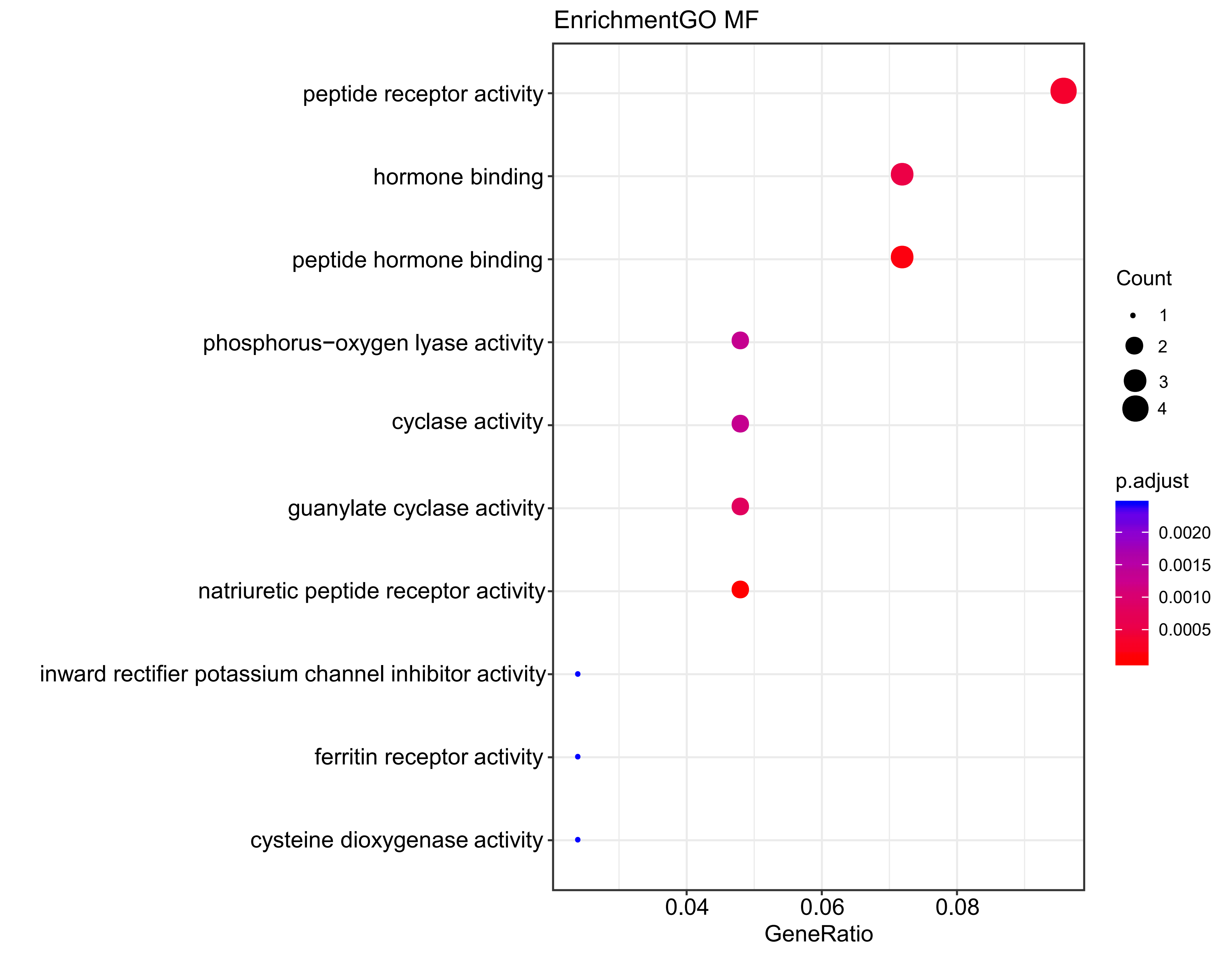

EnrichmentGO MF
peptide receptor activity
hormone binding
peptide hormone binding
phosphorus−oxygen lyase activity
cyclase activity
guanylate cyclase activity
natriuretic peptide receptor activity
inward rectifier potassium channel inhibitor activity
ferritin receptor activity
cysteine dioxygenase activity
0.04
0.06
0.08
GeneRatio
Count
1
2
3
4
p.adjust
0.0020
0.0015
0.0010
0.0005

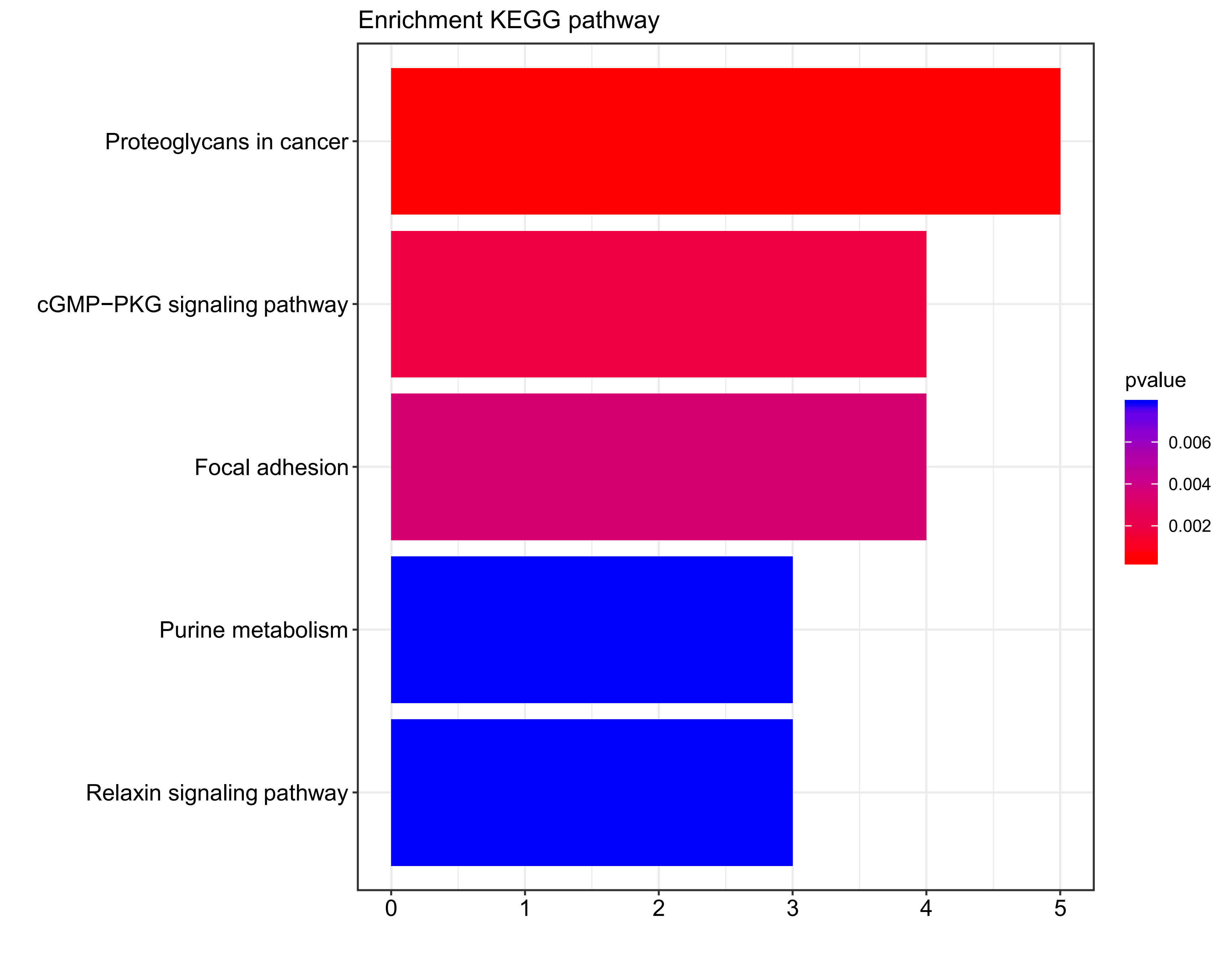
Enrichment KEGG pathway
Proteoglycans in cancer
cGMP−PKG signaling pathway
Focal adhesion
Purine metabolism
Relaxin signaling pathway
0
1
2
3
4
5
pvalue
0.006
0.004
0.002

Figure S3 Bubble diagram of target mRNAs of AC010595.1 in the ceRNA network. We're just listing the results with the smallest p value. **Abbreviations**: GO, gene ontology; KEGG, Kyoto Encyclopedia of Genes and Genomes; BP, biological processes; MF, molecular function; CC, cell component.
