# Supplementary material for: 6-lncRNA Assessment Model for Monitoring and Prognosis of HER2-Positive Breast Cancer: Based on Transcriptome Data
Source: Pathol Oncol Res. 2021 Apr 13;27:609083. doi: 10.3389/pore.2021.609083 (PMC8262145; doi:10.3389/pore.2021.609083)
Supplement: Supplementary file 1 [file DataSheet1.ZIP › Supplementary materials/Fig S4.pdf]

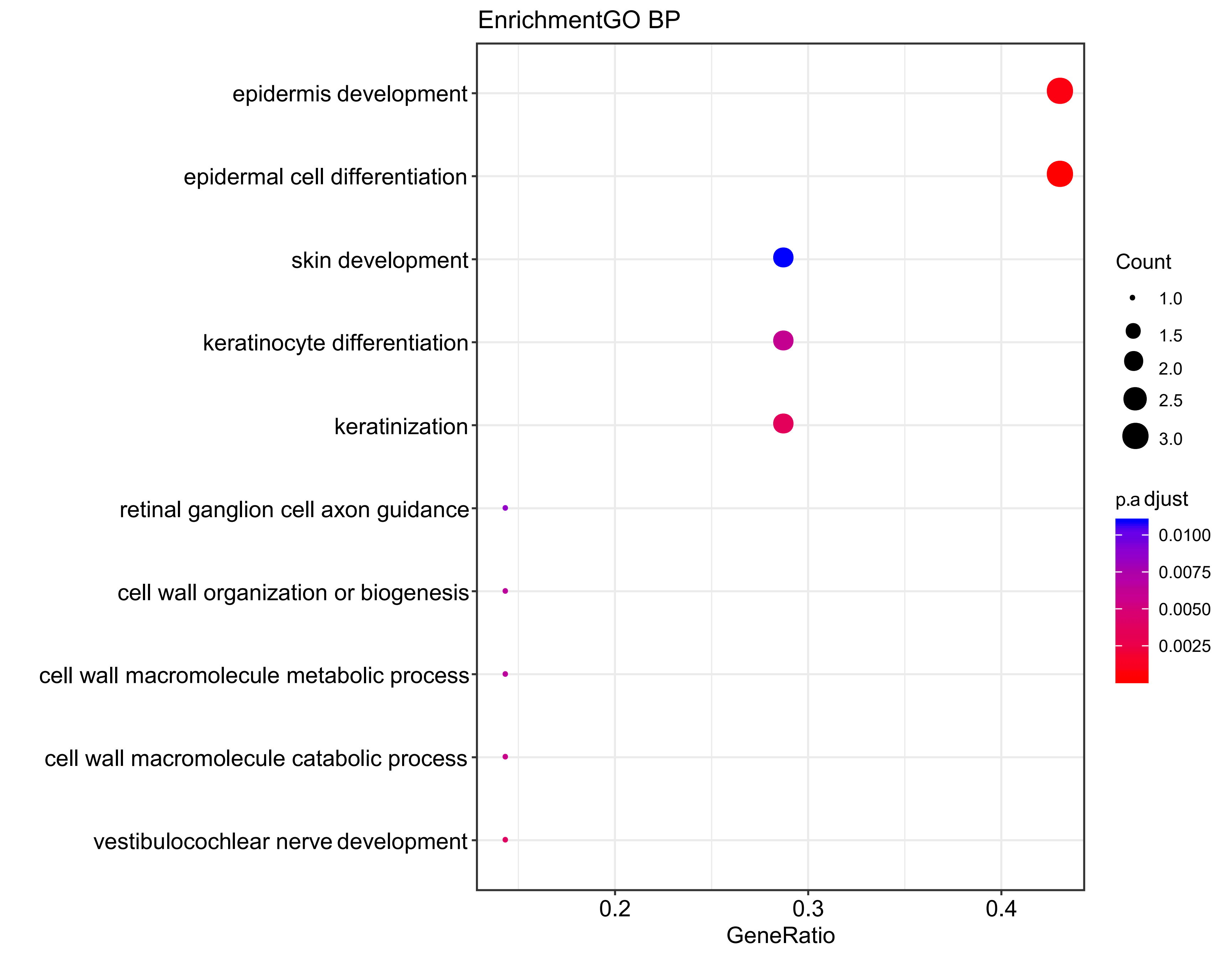
EnrichmentGO BP
epidermis development
epidermal cell differentiation
skin development
keratinocyte differentiation
keratinization
retinal ganglion cell axon guidance
cell wall organization or biogenesis
cell wall macromolecule metabolic process
cell wall macromolecule catabolic process
vestibulocochlear nerve development
0.2
0.3
0.4
GeneRatio
Count
1.0
1.5
2.0
2.5
3.0
p.adjust
0.0100
0.0075
0.0050
0.0025

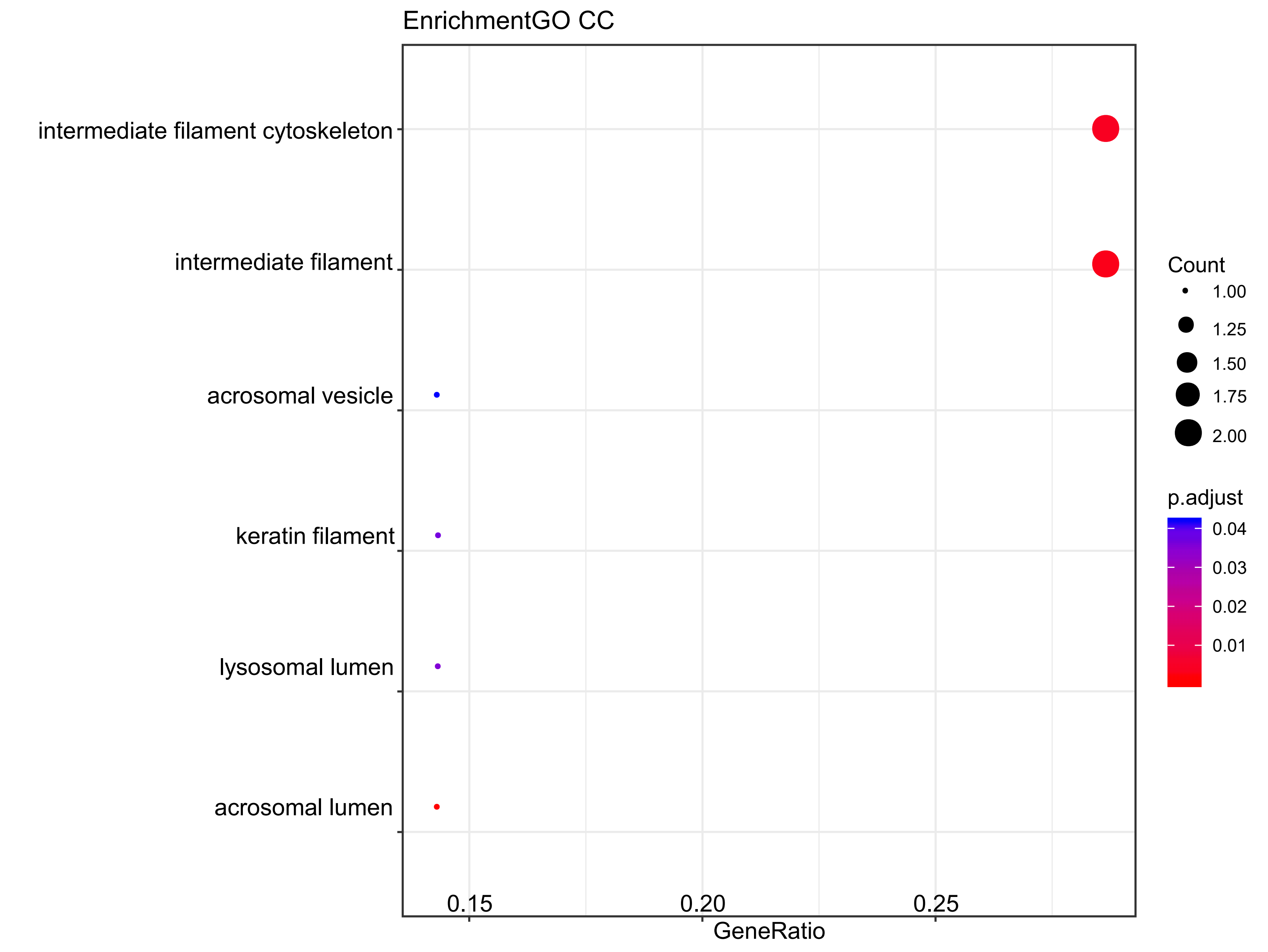
EnrichmentGO CC
intermediate filament cytoskeleton
intermediate filament
acrosomal vesicle
keratin filament
lysosomal lumen
acrosomal lumen
0.15
0.20
0.25
GeneRatio
Count
1.00
1.25
1.50
1.75
2.00
p.adjust
0.04
0.03
0.02
0.01

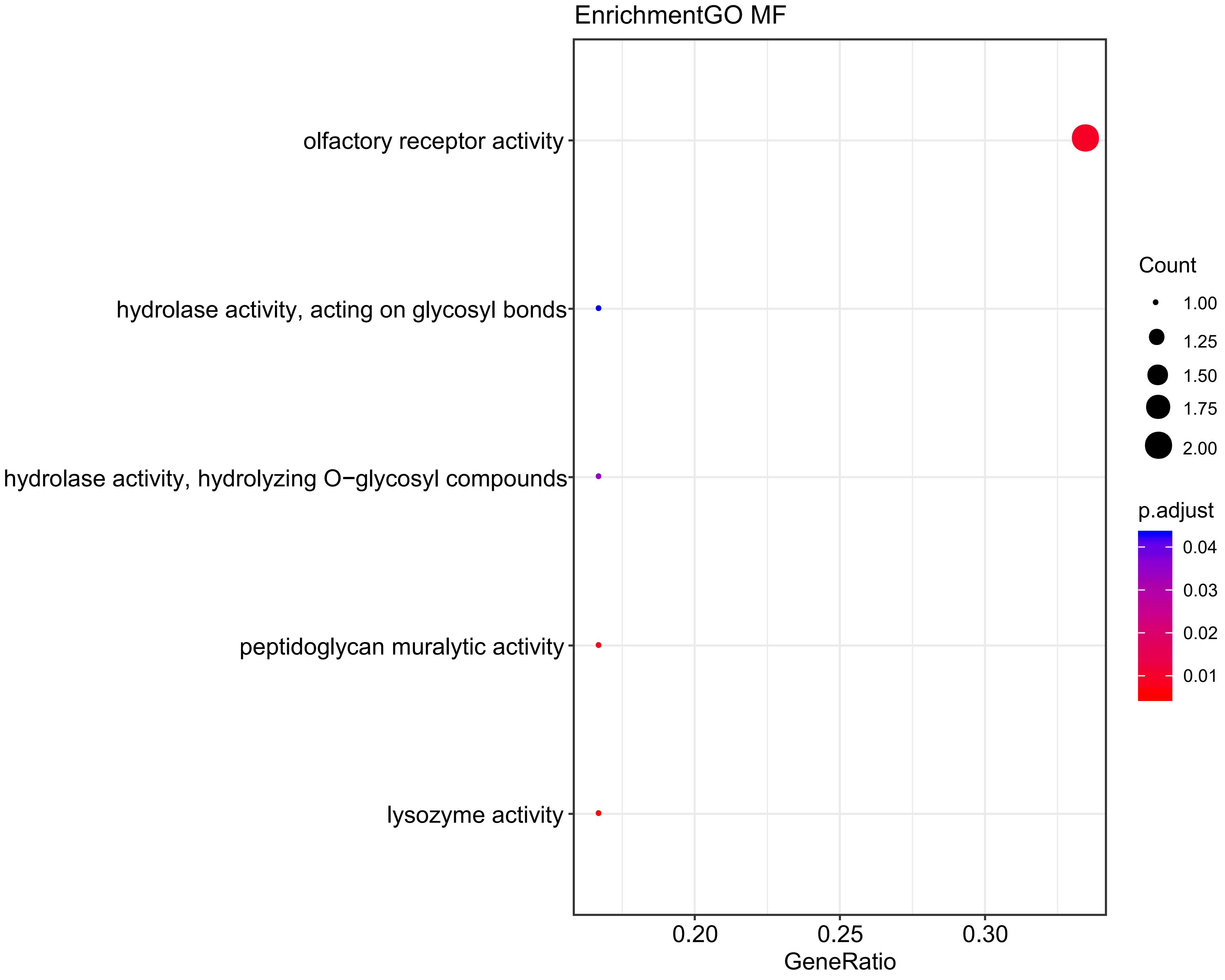
EnrichmentGO MF
olfactory receptor activity
hydrolase activity, acting on glycosyl bonds
hydrolase activity, hydrolyzing O−glycosyl compounds
peptidoglycan muralytic activity
lysozyme activity
0.20
0.25
0.30
GeneRatio
Count
1.00
1.25
1.50
1.75
2.00
p.adjust
0.04
0.03
0.02
0.01

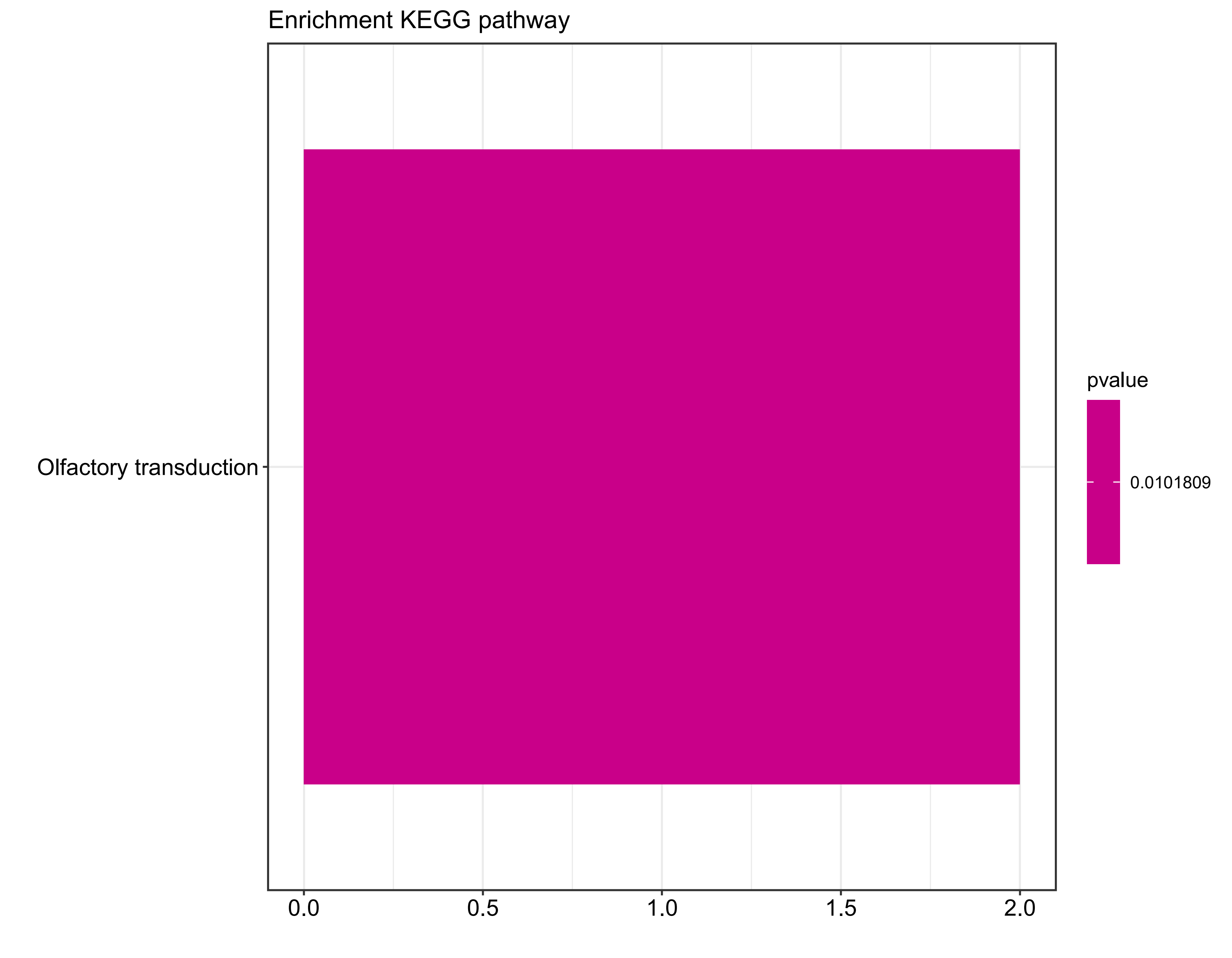

Enrichment KEGG pathway
Olfactory transduction
0.0
0.5
1.0
1.5
2.0
pvalue
0.0101809

Figure S4 Bubble diagram of target mRNAs of AC046168.1 in the ceRNA network. We're just listing the results with the smallest p value. **Abbreviations**: GO, gene ontology; KEGG, Kyoto Encyclopedia of Genes and Genomes; BP, biological processes; MF, molecular function; CC, cell component.
