# Supplementary material for: 6-lncRNA Assessment Model for Monitoring and Prognosis of HER2-Positive Breast Cancer: Based on Transcriptome Data
Source: Pathol Oncol Res. 2021 Apr 13;27:609083. doi: 10.3389/pore.2021.609083 (PMC8262145; doi:10.3389/pore.2021.609083)
Supplement: Supplementary file 1 [file DataSheet1.ZIP › Supplementary materials/Fig S5.pdf]

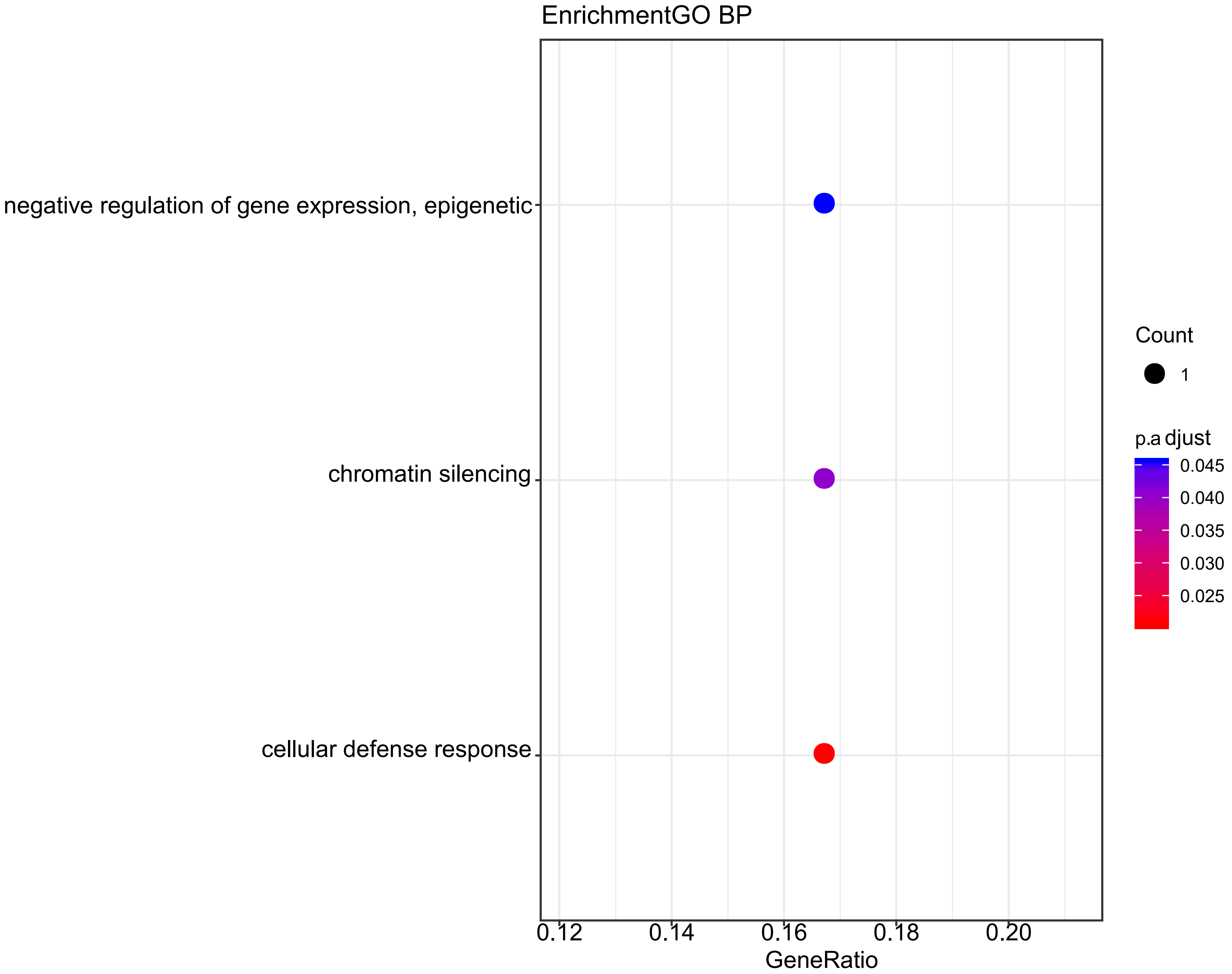
EnrichmentGO BP
negative regulation of gene expression, epigenetic
chromatin silencing
cellular defense response
0.12
0.14
0.16
0.18
0.20
GeneRatio
Count
1
p.adjust
0.045
0.040
0.035
0.030
0.025

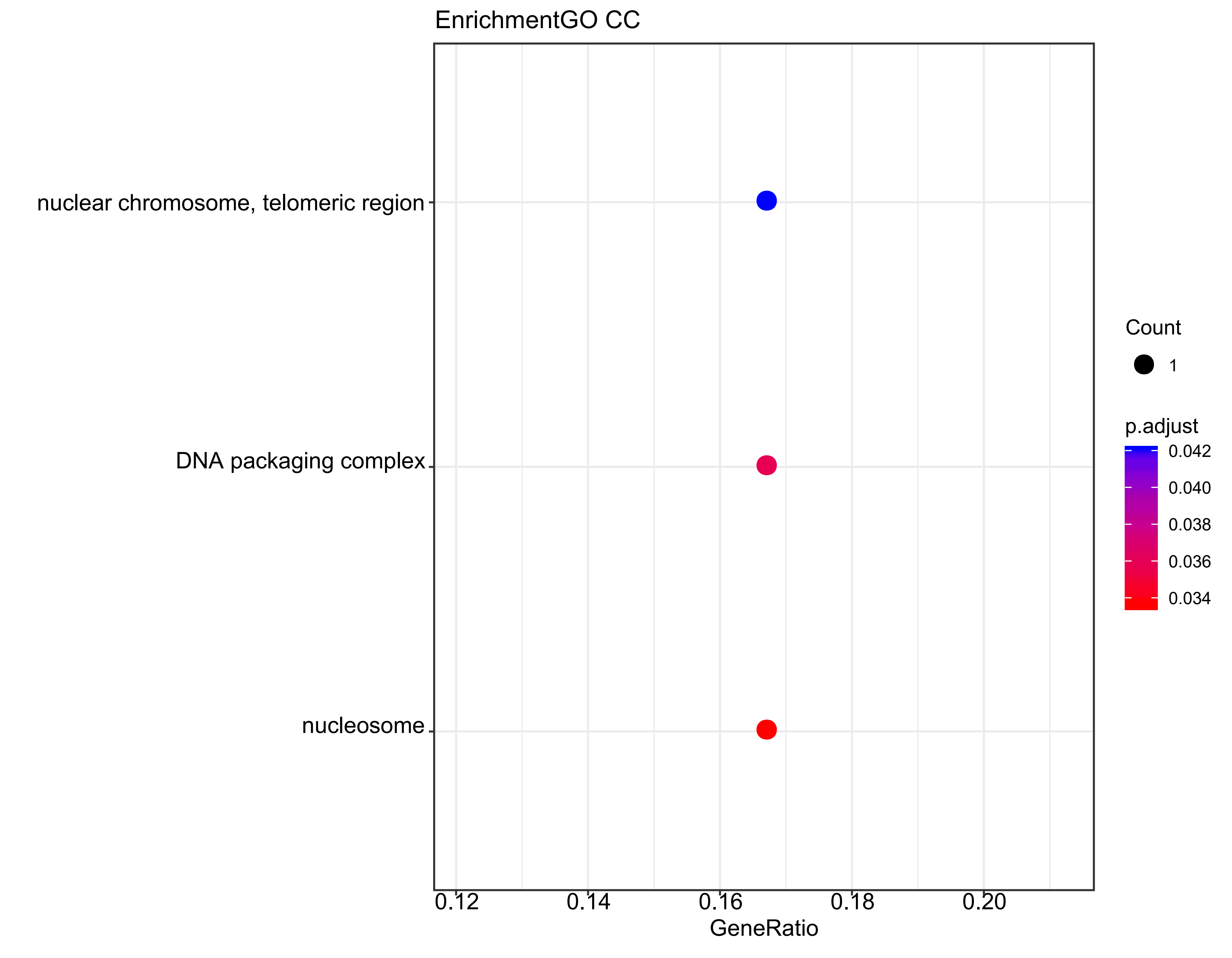
EnrichmentGO CC
nuclear chromosome, telomeric region
DNA packaging complex
nucleosome
0.12
0.14
0.16
0.18
0.20
GeneRatio
Count
1
p.adjust
0.042
0.040
0.038
0.036
0.034

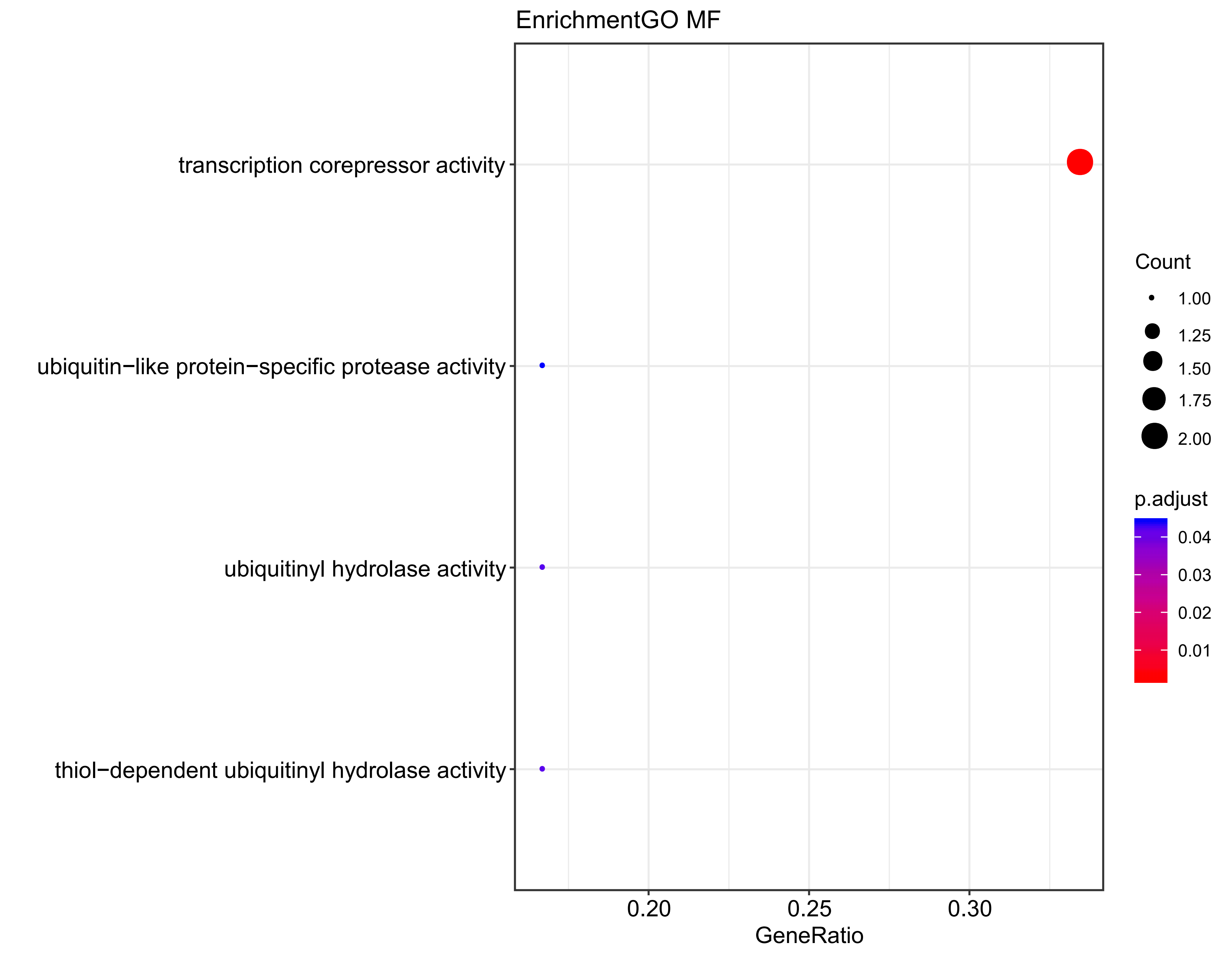
EnrichmentGO MF
transcription corepressor activity
ubiquitin−like protein−specific protease activity
ubiquitinyl hydrolase activity
thiol−dependent ubiquitinyl hydrolase activity
0.20
0.25
0.30
GeneRatio
Count
1.00
1.25
1.50
1.75
2.00
p.adjust
0.04
0.03
0.02
0.01

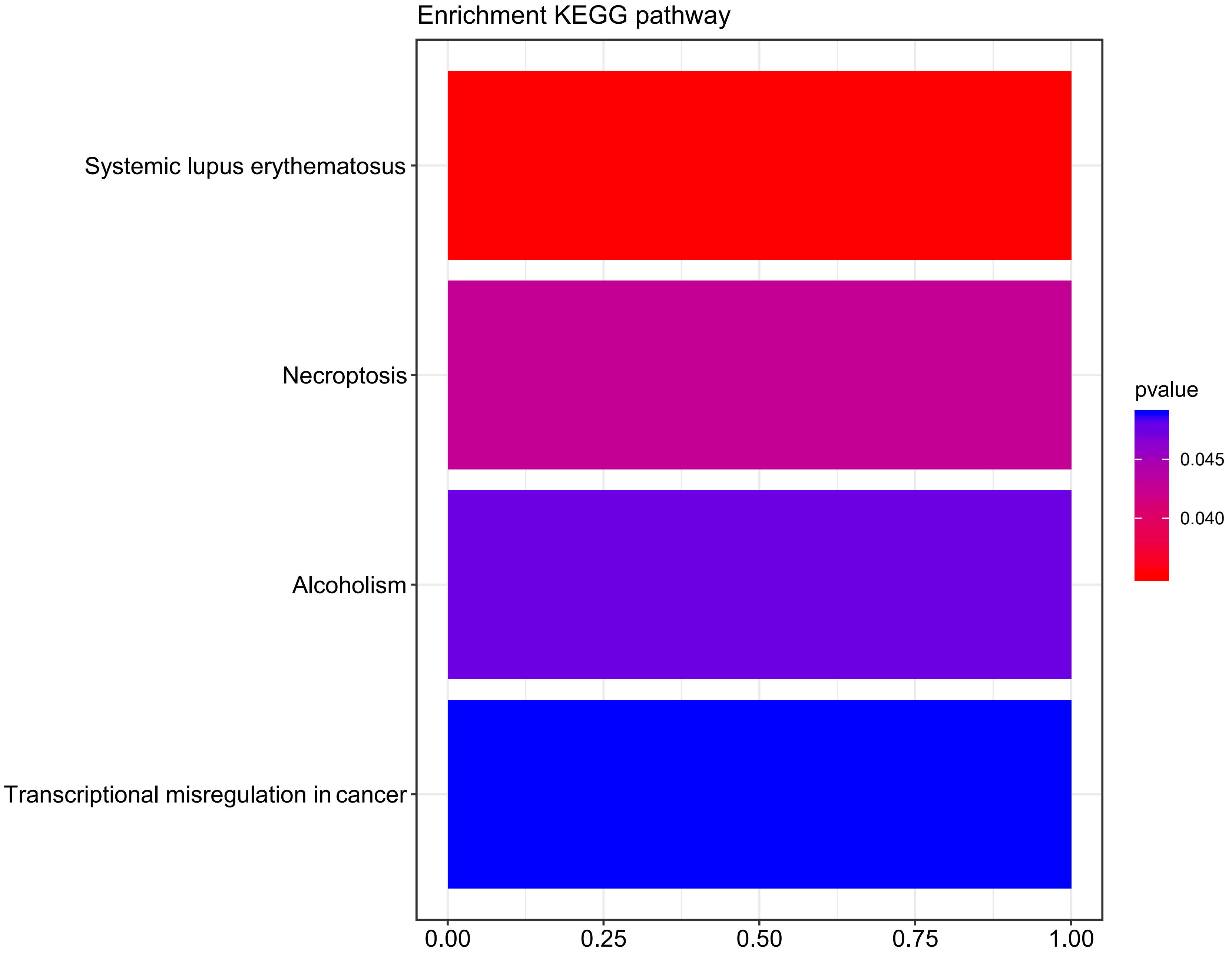
Enrichment KEGG pathway
Systemic lupus erythematosus
Necroptosis
Alcoholism
Transcriptional misregulation in cancer
0.00
0.25
0.50
0.75
1.00
pvalue
0.045
0.040

Figure S5 Bubble diagram of target mRNAs of AC069277.1 in the ceRNA network. We're just listing the results with the smallest p value. **Abbreviations**: GO, gene ontology; KEGG, Kyoto Encyclopedia of Genes and Genomes; BP, biological processes; MF, molecular function; CC, cell component.
