# Supplementary material for: 6-lncRNA Assessment Model for Monitoring and Prognosis of HER2-Positive Breast Cancer: Based on Transcriptome Data
Source: Pathol Oncol Res. 2021 Apr 13;27:609083. doi: 10.3389/pore.2021.609083 (PMC8262145; doi:10.3389/pore.2021.609083)
Supplement: Supplementary file 1 [file DataSheet1.ZIP › Supplementary materials/Fig S7.pdf]

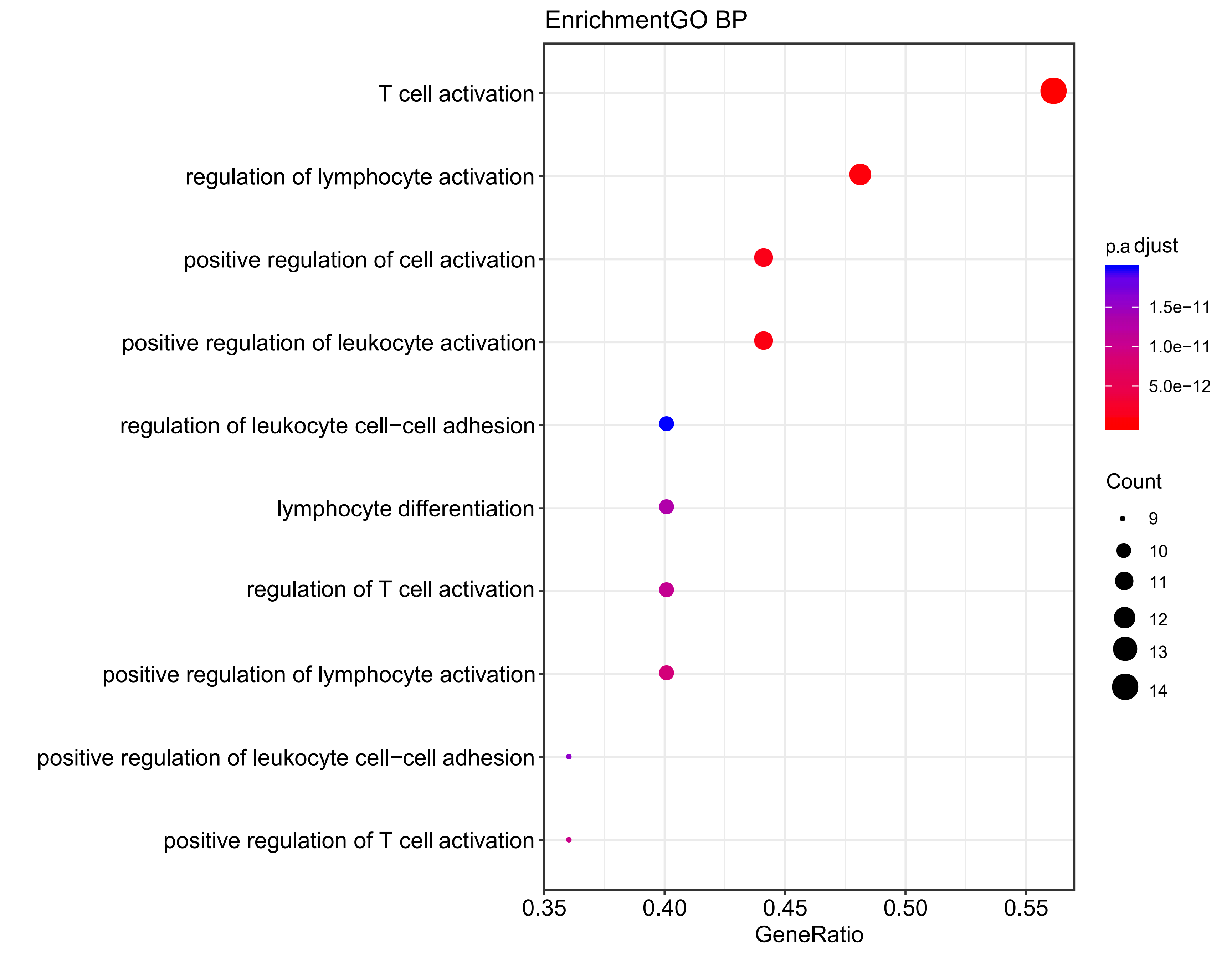
EnrichmentGO BP
T cell activation
regulation of lymphocyte activation
positive regulation of cell activation
positive regulation of leukocyte activation
regulation of leukocyte cell−cell adhesion
lymphocyte differentiation
regulation of T cell activation
positive regulation of lymphocyte activation
positive regulation of leukocyte cell−cell adhesion
positive regulation of T cell activation
0.35
0.40
0.45
0.50
0.55
GeneRatio
p.adjust
1.5e−11
1.0e−11
5.0e−12
Count
9
10
11
12
13
14

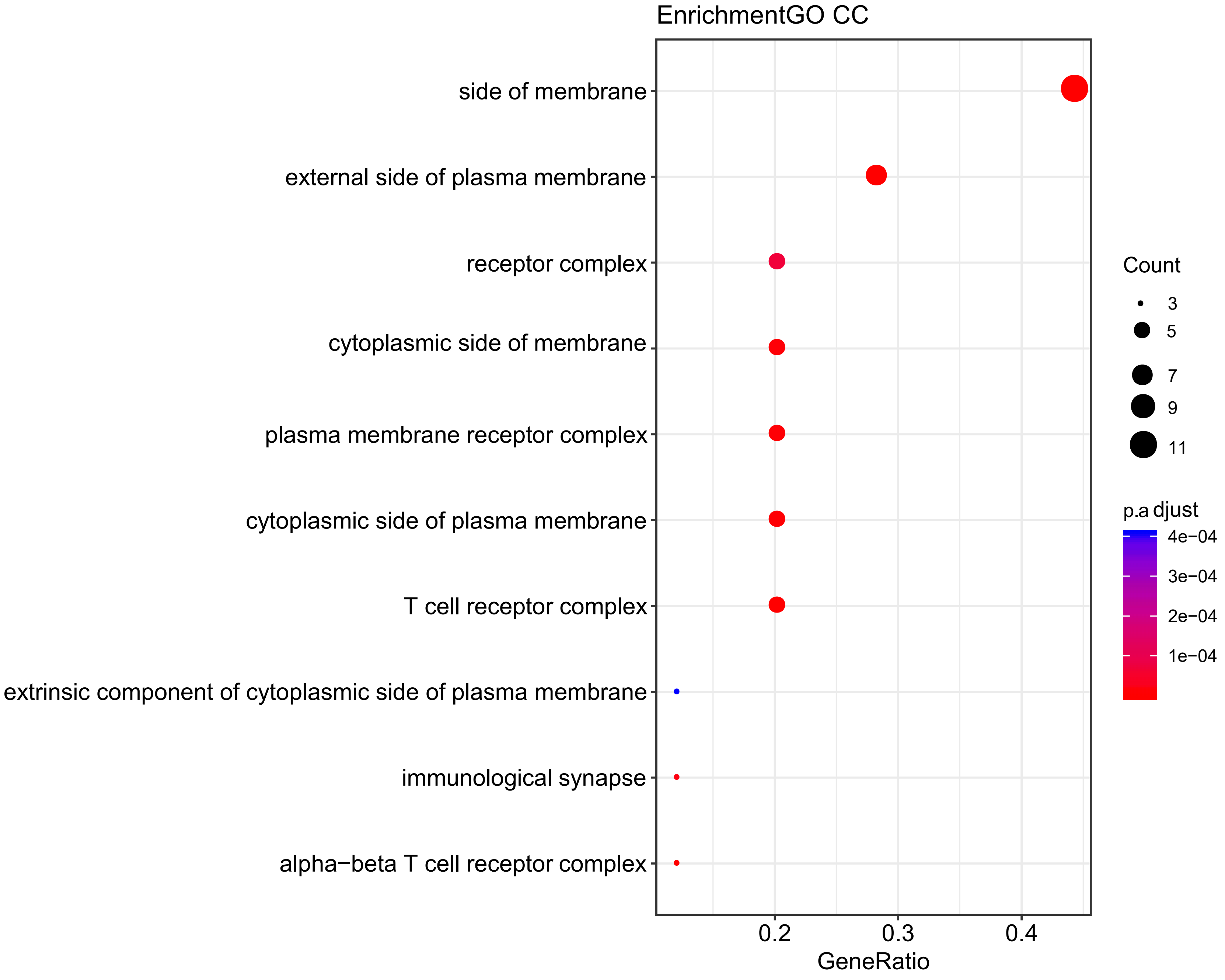
EnrichmentGO CC
side of membrane
external side of plasma membrane
receptor complex
cytoplasmic side of membrane
plasma membrane receptor complex
cytoplasmic side of plasma membrane
T cell receptor complex
extrinsic component of cytoplasmic side of plasma membrane
immunological synapse
alpha−beta T cell receptor complex
0.2
0.3
0.4
GeneRatio
Count
3
5
7
9
11
p.adjust
4e−04
3e−04
2e−04
1e−04

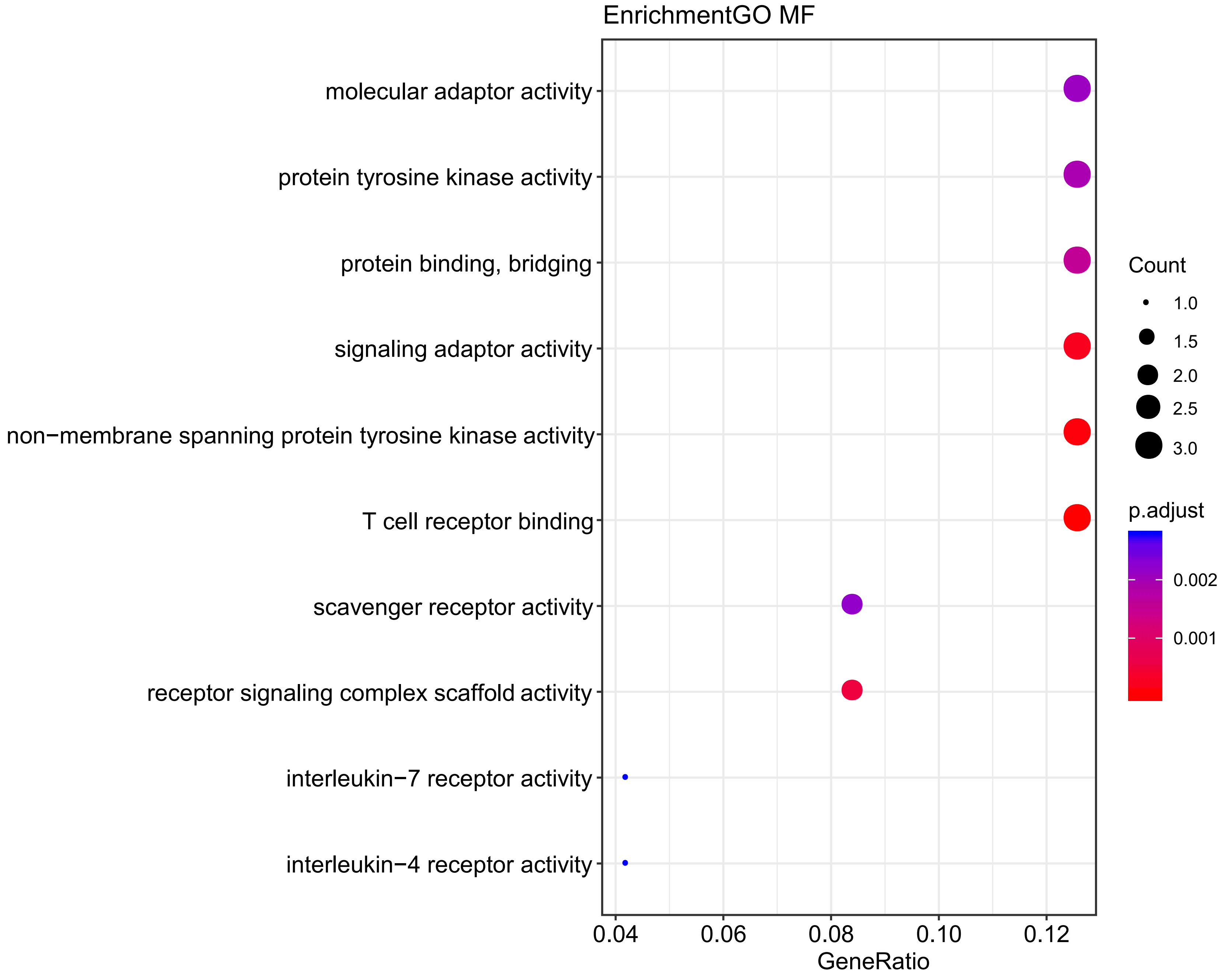

EnrichmentGO MF
molecular adaptor activity
protein tyrosine kinase activity
protein binding, bridging
signaling adaptor activity
non−membrane spanning protein tyrosine kinase activity
T cell receptor binding
scavenger receptor activity
receptor signaling complex scaffold activity
interleukin−7 receptor activity
interleukin−4 receptor activity
0.04
0.06
0.08
0.10
0.12
GeneRatio
Count
1.0
1.5
2.0
2.5
3.0
p.adjust
0.002
0.001

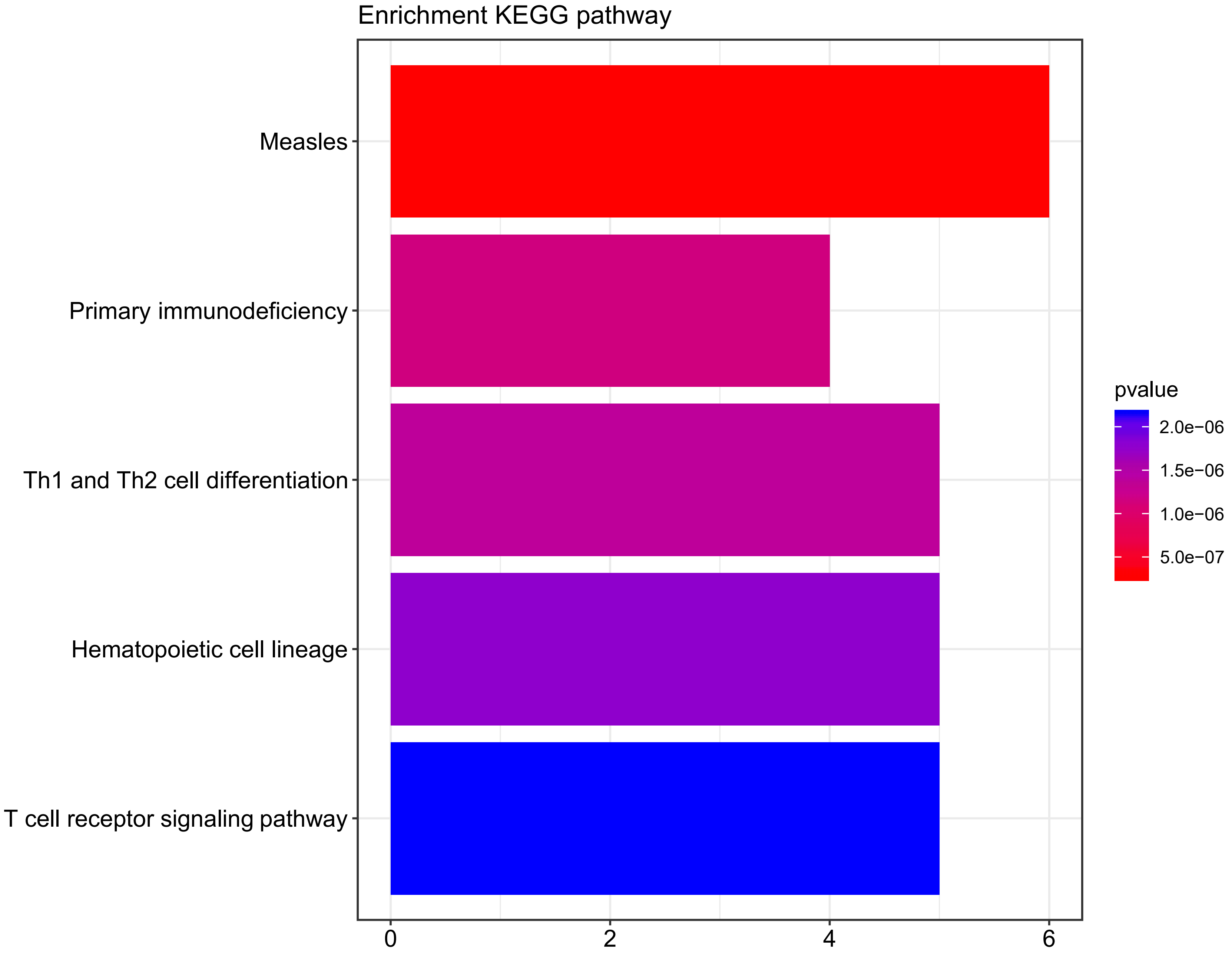
Enrichment KEGG pathway
Measles
Primary immunodeficiency
Th1 and Th2 cell differentiation
Hematopoietic cell lineage
T cell receptor signaling pathway
0
2
4
6
pvalue
2.0e−06
1.5e−06
1.0e−06
5.0e−07

Figure S7 Bubble diagram of target mRNAs of LINC00528 in the ceRNA network. We're just listing the results with the smallest p value. **Abbreviations**: GO, gene ontology; KEGG, Kyoto Encyclopedia of Genes and Genomes; BP, biological processes; MF, molecular function; CC, cell component.
