# Supplementary material for: 6-lncRNA Assessment Model for Monitoring and Prognosis of HER2-Positive Breast Cancer: Based on Transcriptome Data
Source: Pathol Oncol Res. 2021 Apr 13;27:609083. doi: 10.3389/pore.2021.609083 (PMC8262145; doi:10.3389/pore.2021.609083)
Supplement: Supplementary file 1 [file DataSheet1.ZIP › Supplementary materials/Fig S8.pdf]

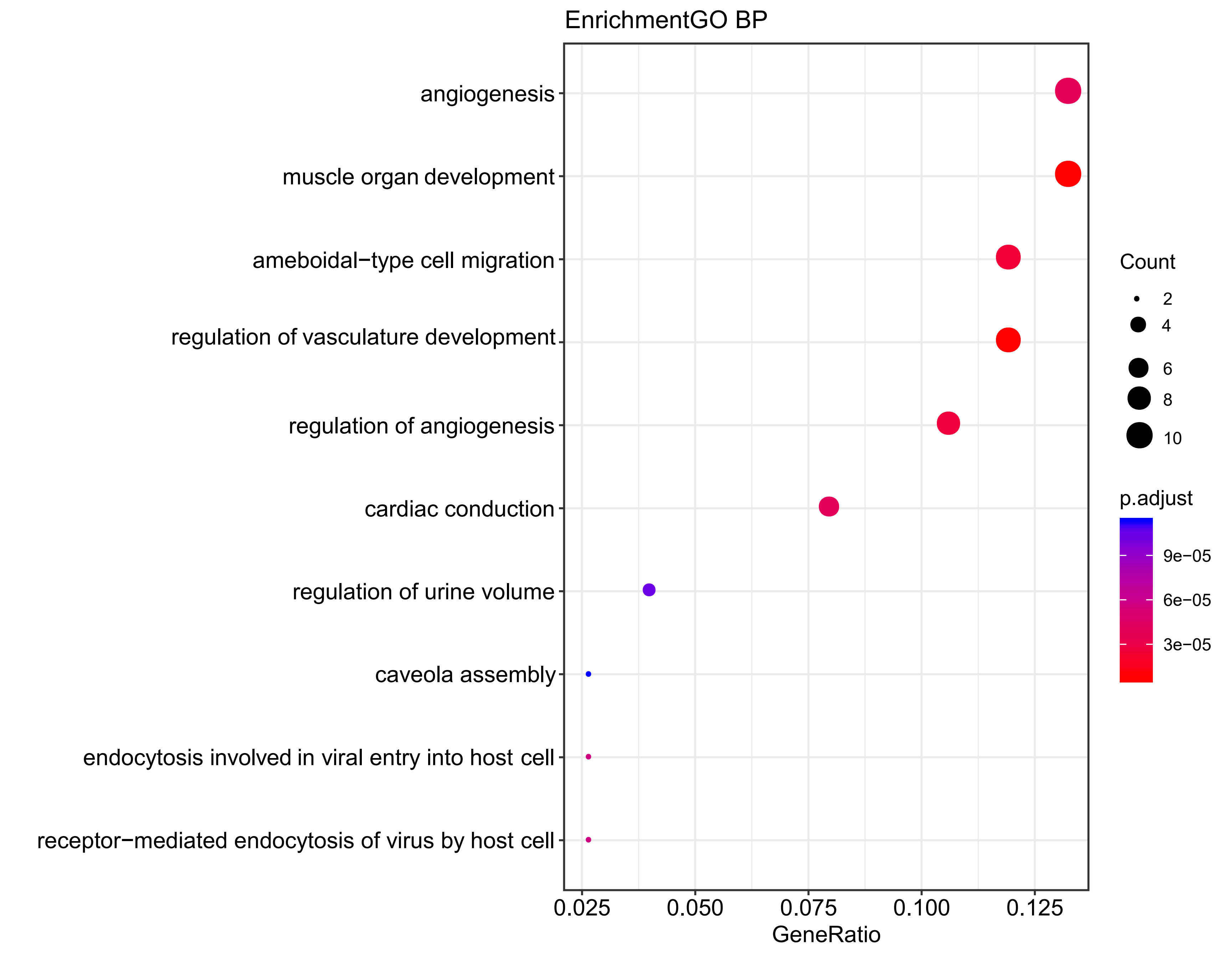
EnrichmentGO BP
angiogenesis
muscle organ development
ameboidal−type cell migration
regulation of vasculature development
regulation of angiogenesis
cardiac conduction
regulation of urine volume
caveola assembly
endocytosis involved in viral entry into host cell
receptor−mediated endocytosis of virus by host cell
0.025
0.050
0.075
0.100
0.125
GeneRatio
Count
2
4
6
8
10
p.adjust
9e−05
6e−05
3e−05

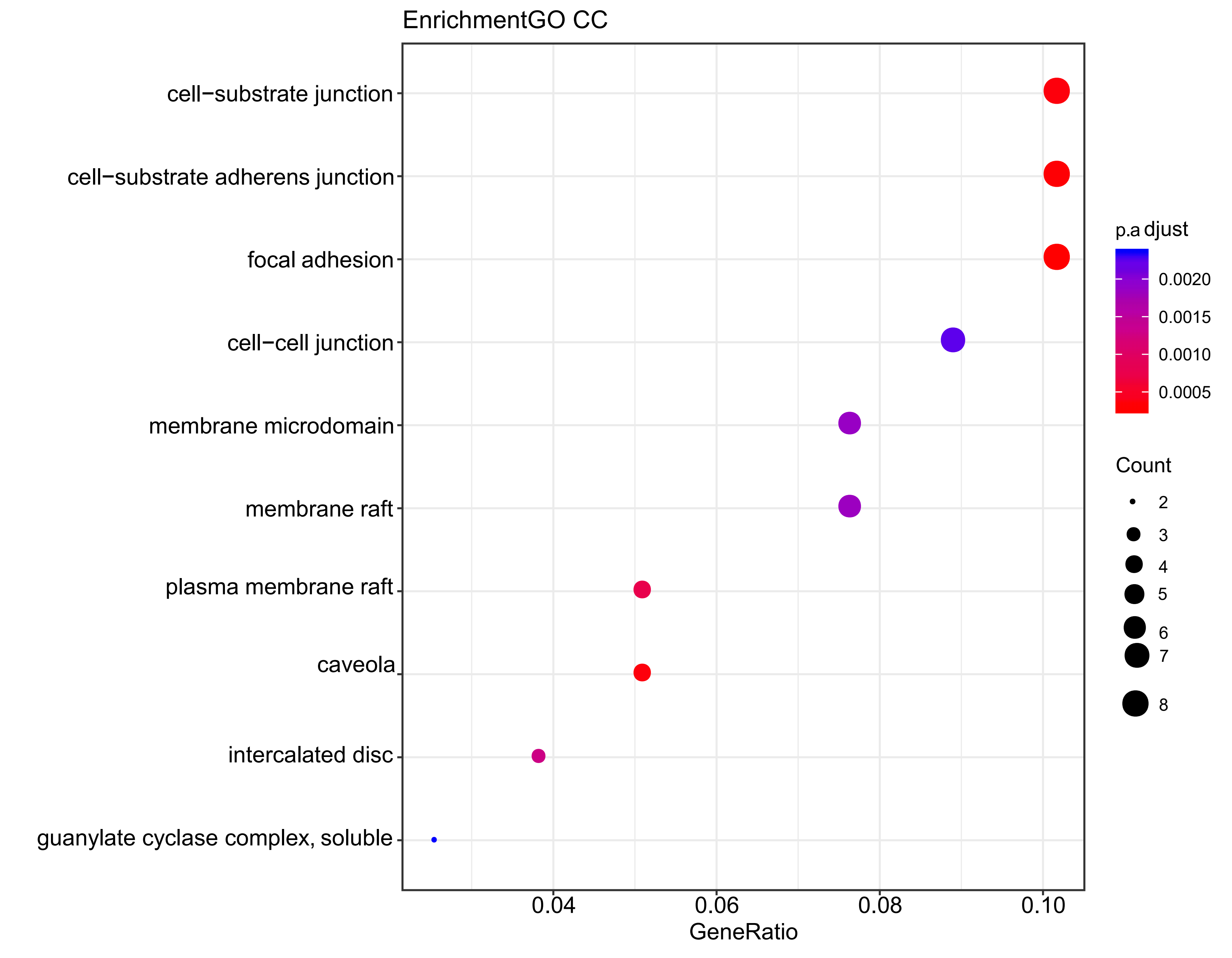
EnrichmentGO CC
cell−substrate junction
cell−substrate adherens junction
focal adhesion
cell−cell junction
membrane microdomain
membrane raft
plasma membrane raft
caveola
intercalated disc
guanylate cyclase complex, soluble
0.04
0.06
0.08
0.10
GeneRatio
p.adjust
0.0020
0.0015
0.0010
0.0005
Count
2
3
4
5
6
7
8

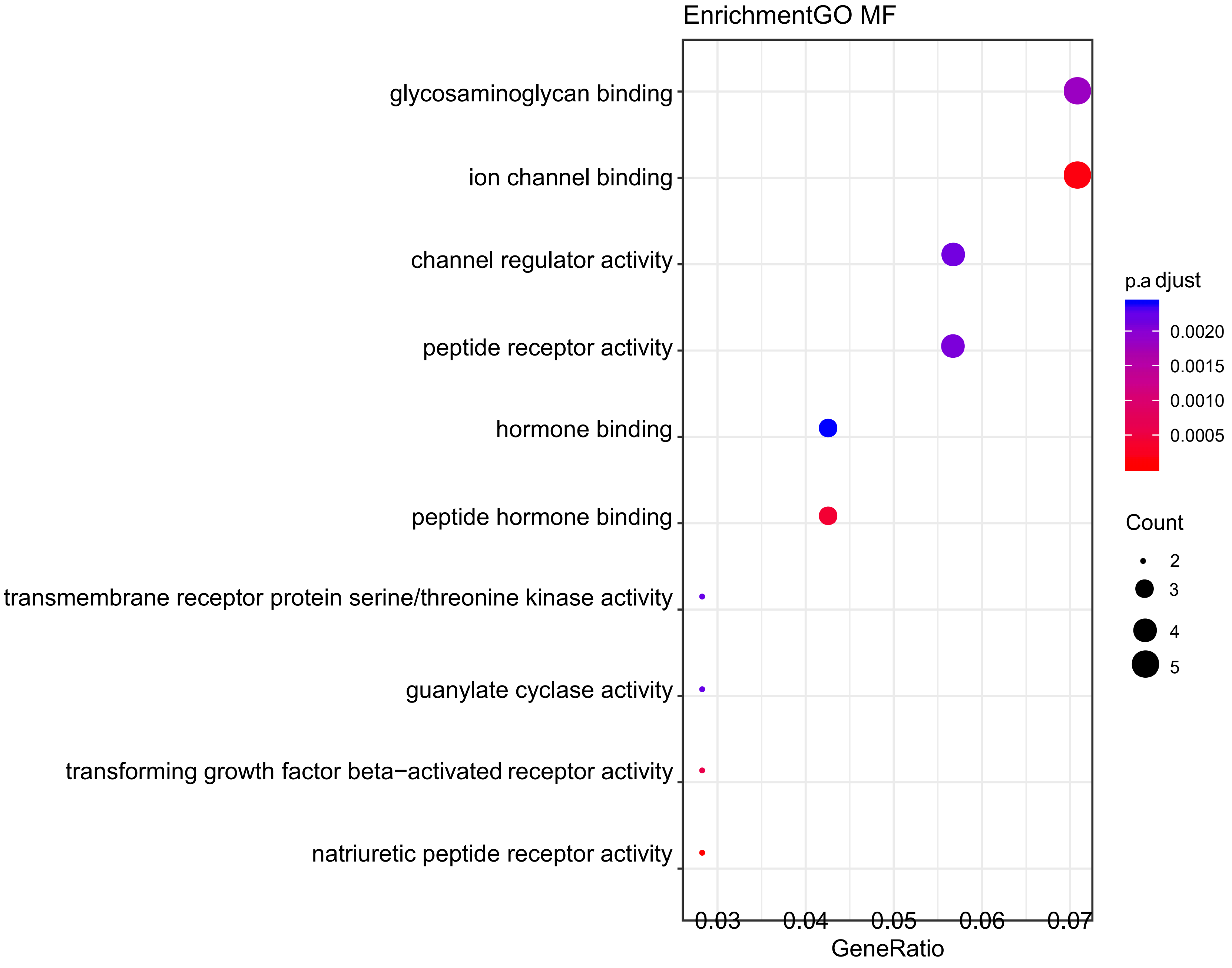
EnrichmentGO MF
glycosaminoglycan binding
ion channel binding
channel regulator activity
peptide receptor activity
hormone binding
peptide hormone binding
transmembrane receptor protein serine/threonine kinase activity
guanylate cyclase activity
transforming growth factor beta−activated receptor activity
natriuretic peptide receptor activity
0.03
0.04
0.05
0.06
0.07
GeneRatio
p.adjust
0.0020
0.0015
0.0010
0.0005
Count
2
3
4
5

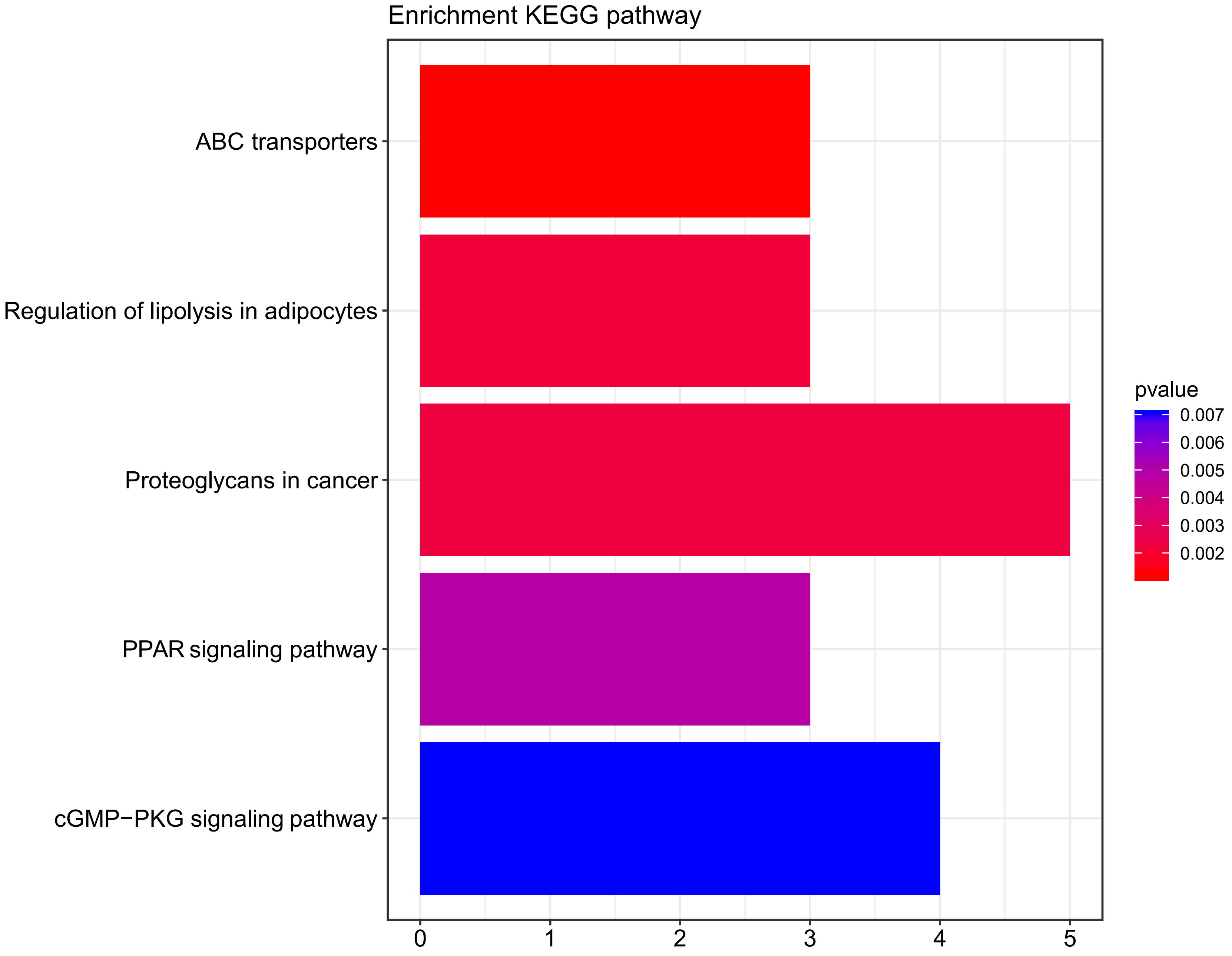

Enrichment KEGG pathway
ABC transporters
Regulation of lipolysis in adipocytes
Proteoglycans in cancer
PPAR signaling pathway
cGMP−PKG signaling pathway
0
1
2
3
4
5
pvalue
0.007
0.006
0.005
0.004
0.003
0.002

Figure S8 Bubble diagram of target mRNAs of MIR762HG in the ceRNA network. We're just listing the results with the smallest p value. **Abbreviations**: GO, gene ontology; KEGG, Kyoto Encyclopedia of Genes and Genomes; BP, biological processes; MF, molecular function; CC, cell component.
