# Supplementary material for: 6-lncRNA Assessment Model for Monitoring and Prognosis of HER2-Positive Breast Cancer: Based on Transcriptome Data
Source: Pathol Oncol Res. 2021 Apr 13;27:609083. doi: 10.3389/pore.2021.609083 (PMC8262145; doi:10.3389/pore.2021.609083)
Supplement: Supplementary file 1 [file Fig S2.pdf › Supplementary materials/Fig S2.pdf]

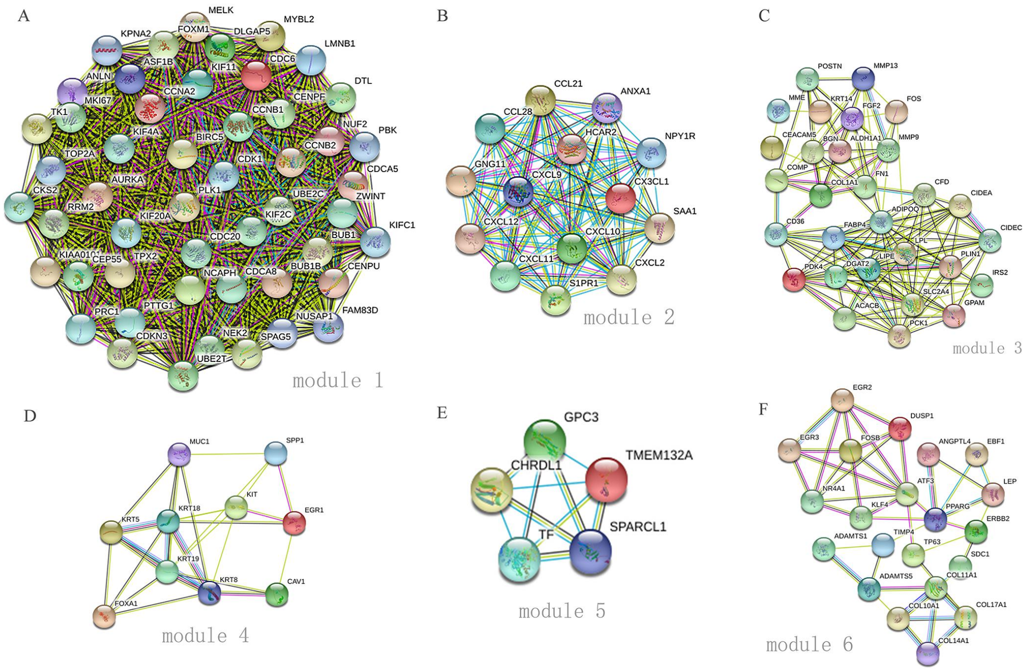

Figure S2 The significant modules in the PPI network. (A) module 1; (B) module 2; (C) module 3; (D)module 4; (E) module 5; (F) module 6. **Abbreviation**: PPI, protein-protein interaction.
