# Supplementary material for: 6-lncRNA Assessment Model for Monitoring and Prognosis of HER2-Positive Breast Cancer: Based on Transcriptome Data
Source: Pathol Oncol Res. 2021 Apr 13;27:609083. doi: 10.3389/pore.2021.609083 (PMC8262145; doi:10.3389/pore.2021.609083)
Supplement: Supplementary file 1 [file DataSheet1.ZIP › Supplementary materials/Table S2.doc]

**Table S2 Module analysis results of the PPI network downloaded from Cytoscape-MCODE**

| Cluster | Score | Nodes | Edges | Node IDs |
| --- | --- | --- | --- | --- |
| 1 | 48.857 | 50 | 1197 | CDC6, KIFC1, UBE2C, PTTG1, CDCA5, TPX2, KIF11, CCNA2, DLGAP5, KIAA0101,  CDCA8, PLK1, AURKA, CCNB2, CEP55, NUF2, NCAPH, BUB1, CDKN3, CDC20, CDK1, ZWINT, CCNB1, CKS2, BUB1B, NEK2, KIF4A, UBE2T, DTL, CENPU, PBK, TK1, TOP2A, NUSAP1, SPAG5, FOXM1, KIF20A, ASF1B, RRM2, ANLN, MELK, KIF2C, FAM83D, LMNB1, CENPF, KPNA2, MYBL2, PRC1, MKI67, BIRC5 |
|  |  |  |  |  |
|  |  |  |  |  |
|  |  |  |  |  |
|  |  |  |  |  |
| 2 | 14 | 14 | 91 | HCAR2, NPY1R, CXCL2, CCL28, CCL21, CXCL12, S1PR1, CXCL11, CXCL10, GNG11, CX3CL1, CXCL9, ANXA1, SAA1 |
|  |  |  |  |  |
| 3 | 9.357 | 29 | 131 | BGN, ADIPOQ, SLC2A4, COL1A1, COMP, PDK4, MMP9, PCK1, IRS2, LIPE, FGF2,  LPL, PLIN1, CEACAM5, GPAM, FABP4, CFD, FN1, ALDH1A1, FOS, CD36,  MMP13, KRT14, ACACB, DGAT2, CIDEA, CIDEC, POSTN, MME |
|  |  |  |  |  |
|  |  |  |  |  |
| 4 | 6 | 10 | 27 | MUC1, KRT19, SPP1, KIT, EGR1, KRT5, CAV1, FOXA1, KRT18, KRT8 |
| 5 | 5 | 5 | 10 | GPC3, TF, CHRDL1, TMEM132A, SPARCL1 |
| 6 | 4.286 | 22 | 45 | TP63, NR4A1, COL11A1, DUSP1, TNS1, PPARG, ERBB2, LEP, FOSB, EBF1,  ADAMTS1, ADAMTS5, COL17A1, COL14A1, ANGPTL4, TIMP4, SDC1, EGR2, ATF3, COL10A1, EGR3, KLF4 |
|  |  |  |  |  |
|  |  |  |  |  |

**Abbreviations:** PPI: protein-protein interaction.
