# Supplementary material for: 6-lncRNA Assessment Model for Monitoring and Prognosis of HER2-Positive Breast Cancer: Based on Transcriptome Data
Source: Pathol Oncol Res. 2021 Apr 13;27:609083. doi: 10.3389/pore.2021.609083 (PMC8262145; doi:10.3389/pore.2021.609083)
Supplement: Supplementary file 1 [file DataSheet1.ZIP › Supplementary materials/Table S3.doc]

**Table S3 The function analysis results of each module**

| Term ID | Term description | Count | FDR |  |
| --- | --- | --- | --- | --- |
| **Module 1** | | | | |
| **BP** |  |  |  |  |
| GO:0000278 | mitotic cell cycle | 38 | 5.10E-43 |  |
| GO:1903047 | mitotic cell cycle process | 37 | 5.10E-43 |  |
| GO:0022402 | cell cycle process | 40 | 1.22E-41 |  |
| GO:0007049 | cell cycle | 43 | 2.44E-41 |  |
| GO:0051301 | cell division | 34 | 5.41E-40 |  |
| **CC** |  |  |  |  |
| GO:0005819 | spindle | 21 | 5.32E-22 |  |
| GO:0015630 | microtubule cytoskeleton | 27 | 9.89E-19 |  |
| GO:0044430 | cytoskeletal part | 29 | 1.08E-17 |  |
| GO:0000793 | condensed chromosome | 16 | 1.29E-17 |  |
| GO:0000775 | chromosome, centromeric region | 15 | 6.47E-17 |  |
| **MF** |  |  |  |  |
| GO:0008017 | microtubule binding | 11 | 9.56E-09 |  |
| GO:0005524 | ATP binding | 20 | 1.61E-08 |  |
| GO:0019900 | kinase binding | 13 | 2.42E-07 |  |
| GO:0019899 | enzyme binding | 21 | 4.90E-07 |  |
| GO:0019901 | protein kinase binding | 12 | 4.90E-07 |  |
| **KEGG** |  |  |  |  |
| hsa04110 | Cell cycle | 10 | 3.21E-11 |  |
| hsa04114 | Oocyte meiosis | 8 | 1.18E-08 |  |
| hsa04914 | Progesterone-mediated oocyte maturation | 7 | 5.83E-08 |  |
| hsa04218 | Cellular senescence | 6 | 2.30E-05 |  |
| hsa04115 | p53 signaling pathway | 4 | 0.00018 |  |
| **Reactome** |  |  |  |  |
| HSA-69278 | Cell Cycle, Mitotic | 29 | 4.54E-31 |  |
| HSA-68886 | M Phase | 20 | 2.53E-20 |  |
| HSA-68877 | Mitotic Prometaphase | 16 | 2.22E-18 |  |
| HSA-2500257 | Resolution of Sister Chromatid Cohesion | 14 | 5.97E-18 |  |
| HSA-69620 | Cell Cycle Checkpoints | 16 | 2.21E-16 |  |
| **Module 2** | | | | |
| **BP** |  |  |  |  |
| GO:0060326 | cell chemotaxis | 11 | 2.49E-17 |  |
| GO:0007186 | G protein-coupled receptor signaling pathway | 14 | 1.02E-14 |  |
| GO:0072676 | lymphocyte migration | 8 | 1.59E-14 |  |
| GO:0030595 | leukocyte chemotaxis | 9 | 1.77E-14 |  |
| GO:0048247 | lymphocyte chemotaxis | 7 | 3.06E-13 |  |
| **CC** |  |  |  |  |
| GO:0005615 | extracellular space | 9 | 1.31E-06 |  |
| GO:0009897 | external side of plasma membrane | 5 | 1.23E-05 |  |
| GO:0098552 | side of membrane | 6 | 1.23E-05 |  |
| GO:0005576 | extracellular region | 10 | 1.65E-05 |  |
| GO:0009986 | cell surface | 6 | 8.67E-05 |  |
| **MF** |  |  |  |  |
| GO:0008009 | chemokine activity | 8 | 6.14E-16 |  |
| GO:0001664 | G protein-coupled receptor binding | 10 | 8.10E-15 |  |
| GO:0045236 | CXCR chemokine receptor binding | 5 | 2.64E-11 |  |
| GO:0048018 | receptor ligand activity | 9 | 5.42E-11 |  |
| GO:0005102 | signaling receptor binding | 11 | 1.55E-09 |  |
| **KEGG** |  |  |  |  |
| hsa04062 | Chemokine signaling pathway | 9 | 4.36E-14 |  |
| hsa04060 | Cytokine-cytokine receptor interaction | 8 | 6.11E-11 |  |
| hsa04064 | NF-kappa B signaling pathway | 3 | 0.00048 |  |
| hsa04620 | Toll-like receptor signaling pathway | 3 | 0.00048 |  |
| hsa04668 | TNF signaling pathway | 3 | 0.00048 |  |
| **Reactome** |  |  |  |  |
| HSA-418594 | G alpha (i) signalling events | 14 | 1.73E-22 |  |
| HSA-500792 | GPCR ligand binding | 14 | 5.56E-22 |  |
| HSA-373076 | Class A/1 (Rhodopsin-like receptors) | 13 | 2.40E-21 |  |
| HSA-375276 | Peptide ligand-binding receptors | 11 | 5.72E-19 |  |
| HSA-380108 | Chemokine receptors bind chemokines | 8 | 1.07E-16 |  |
| **Module 3** | | | | |
| **BP** |  |  |  |  |
| GO:0010033 | response to organic substance | 20 | 7.10E-08 |  |
| GO:0034097 | response to cytokine | 14 | 7.10E-08 |  |
| GO:0006629 | lipid metabolic process | 14 | 1.77E-07 |  |
| GO:0071345 | cellular response to cytokine stimulus | 13 | 1.77E-07 |  |
| GO:0006641 | triglyceride metabolic process | 6 | 3.97E-07 |  |
| **CC** |  |  |  |  |
| GO:0005811 | lipid droplet | 6 | 3.02E-07 |  |
| GO:0031012 | extracellular matrix | 8 | 6.30E-07 |  |
| GO:0044421 | extracellular region part | 11 | 0.00013 |  |
| GO:0005576 | extracellular region | 13 | 0.001 |  |
| GO:0005615 | extracellular space | 9 | 0.001 |  |
| **MF** |  |  |  |  |
| GO:0005518 | collagen binding | 4 | 0.00019 |  |
| GO:0005539 | glycosaminoglycan binding | 6 | 0.00019 |  |
| GO:1901681 | sulfur compound binding | 6 | 0.00019 |  |
| GO:0008201 | heparin binding | 5 | 0.00024 |  |
| GO:0044877 | protein-containing complex binding | 8 | 0.0029 |  |
| **KEGG** |  |  |  |  |
| hsa04152 | AMPK signaling pathway | 7 | 5.13E-08 |  |
| hsa03320 | PPAR signaling pathway | 6 | 5.17E-08 |  |
| hsa04920 | Adipocytokine signaling pathway | 6 | 5.17E-08 |  |
| hsa04931 | Insulin resistance | 5 | 1.35E-05 |  |
| hsa04910 | Insulin signaling pathway | 5 | 2.63E-05 |  |
| **Reactome** |  |  |  |  |
| HSA-381340 | Transcriptional regulation of white adipocyte | 7 | 1.29E-08 |  |
|  | differentiation |  |  |  |
| HSA-8979227 | Triglyceride metabolism | 5 | 5.34E-07 |  |
| HSA-1430728 | Metabolism | 15 | 3.01E-06 |  |
| HSA-1266738 | Developmental Biology | 11 | 7.95E-06 |  |
| HSA-556833 | Metabolism of lipids | 9 | 3.56E-05 |  |
| **Module 4** | | | | |
| **BP** |  |  |  |  |
| GO:0009888 | tissue development | 8 | 0.00012 |  |
| GO:0048513 | animal organ development | 9 | 0.00016 |  |
| GO:0070268 | cornification | 4 | 0.00016 |  |
| GO:0007166 | cell surface receptor signaling pathway | 8 | 0.0003 |  |
| GO:0030154 | cell differentiation | 9 | 0.0003 |  |
| **CC** |  |  |  |  |
| GO:0005882 | intermediate filament | 4 | 0.00029 |  |
| GO:0045095 | keratin filament | 3 | 0.00067 |  |
| GO:0016010 | dystrophin-associated glycoprotein complex | 2 | 0.0018 |  |
| GO:0016327 | apicolateral plasma membrane | 2 | 0.0018 |  |
| GO:0043034 | costamere | 2 | 0.0018 |  |
| **MF** |  |  |  |  |
| GO:0097110 | scaffold protein binding | 3 | 0.00037 |  |
| GO:0005198 | structural molecule activity | 5 | 0.00063 |  |
| GO:0005515 | protein binding | 9 | 0.015 |  |
| GO:0005200 | structural constituent of cytoskeleton | 2 | 0.0372 |  |
| **Reactome** |  |  |  |  |
| HSA-6809371 | Formation of the cornified envelope | 4 | 3.01E-05 |  |
| HSA-9006934 | Signaling by Receptor Tyrosine Kinases | 3 | 0.0301 |  |
| **Module 5** | | | | |
| **BP** |  |  |  |  |
| GO:0043687 | post-translational protein modification | 5 | 7.50E-07 |  |
| GO:0030510 | regulation of BMP signaling pathway | 2 | 0.0136 |  |
| GO:0045807 | positive regulation of endocytosis | 2 | 0.02 |  |
| GO:0001503 | ossification | 2 | 0.0424 |  |
| **CC** |  |  |  |  |
| GO:0005788 | endoplasmic reticulum lumen | 5 | 8.23E-08 |  |
| GO:0062023 | collagen-containing extracellular matrix | 2 | 0.0128 |  |
| GO:0005576 | extracellular region | 4 | 0.019 |  |
| **Reactome** |  |  |  |  |
| HSA-8957275 | Post-translational protein phosphorylation | 5 | 2.20E-10 |  |
| HSA-381426 | Regulation of Insulin-like Growth Factor (IGF) | 5 | 2.27E-10 |  |
|  | transport and uptake by Insulin-like Growth |  |  |  |
|  | Factor Binding Proteins (IGFBPs) |  |  |  |
| **Module 6** | | | | |
| **BP** |  |  |  |  |
| GO:0048856 | anatomical structure development | 19 | 1.49E-06 |  |
| GO:0007275 | multicellular organism development | 18 | 2.48E-06 |  |
| GO:0048646 | anatomical structure formation involved in morphogenesis | 10 | 2.48E-06 |  |
| GO:0009653 | anatomical structure morphogenesis | 13 | 3.87E-06 |  |
| GO:0009719 | response to endogenous stimulus | 11 | 8.83E-06 |  |
| **CC** |  |  |  |  |
| GO:0031012 | extracellular matrix | 6 | 5.27E-05 |  |
| GO:0062023 | collagen-containing extracellular matrix | 5 | 5.27E-05 |  |
| GO:0005581 | collagen trimer | 4 | 0.0001 |  |
| GO:0044421 | extracellular region part | 9 | 0.00017 |  |
| GO:0005788 | endoplasmic reticulum lumen | 5 | 0.00035 |  |
| **MF** |  |  |  |  |
| GO:0001228 | DNA-binding transcription activator activity, | 6 | 0.00064 |  |
|  | RNA polymerase II-specific |  |  |  |
| GO:0005515 | protein binding | 17 | 0.00064 |  |
| GO:0043565 | sequence-specific DNA binding | 8 | 0.00064 |  |
| GO:0000981 | DNA-binding transcription factor activity, | 9 | 0.00083 |  |
|  | RNA polymerase II-specific |  |  |  |
| GO:0000978 | RNA polymerase II proximal promoter sequence-specific | 5 | 0.0027 |  |
|  | DNA binding |  |  |  |
| **KEGG** |  |  |  |  |
| hsa04974 | Protein digestion and absorption | 4 | 0.00015 |  |
| **Reactome** |  |  |  |  |
| HSA-381340 | Transcriptional regulation of white adipocyte | 6 | 5.14E-08 |  |
|  | differentiation |  |  |  |
| HSA-1474228 | Degradation of the extracellular matrix | 6 | 4.96E-07 |  |
| HSA-1474244 | Extracellular matrix organization | 7 | 8.99E-07 |  |
| HSA-8948216 | Collagen chain trimerization | 4 | 6.22E-06 |  |
| HSA-2022090 | Assembly of collagen fibrils and other | 4 | 1.61E-05 |  |
|  | multimeric structures |  |  |  |

**Abbreviations:** FDR: False discovery rate; BP: biology process; CC: cell component; MF: molecular function; GO: gene ontology; KEGG: Kyoto Encyclopedia of Genes and Genomes.
