# Supplementary material for: 6-lncRNA Assessment Model for Monitoring and Prognosis of HER2-Positive Breast Cancer: Based on Transcriptome Data
Source: Pathol Oncol Res. 2021 Apr 13;27:609083. doi: 10.3389/pore.2021.609083 (PMC8262145; doi:10.3389/pore.2021.609083)
Supplement: Supplementary file 1 [file DataSheet1.ZIP › Supplementary materials/Table S4.doc]

**Table S4 Connectivity analysis results of the PPI network downloaded from Cytoscape-Centiscape.**

| Name | Betweenness unDir | Bridging unDir | Degree unDir | MCODE_Cluster | MCODE_Score |
| --- | --- | --- | --- | --- | --- |
| FN1 | 19906.61137 | 29.9536039 | 86 | Cluster 3 | 8.571428571 |
| EGFR | 14038.31522 | 21.79719279 | 75 | Cluster 12 | 4.40285205 |
| CDK1 | 3095.998876 | 17.70445222 | 65 | Cluster 1 | 43.43877551 |
| MKI67 | 5444.20968 | 59.51698707 | 63 | Cluster 1 | 43.43877551 |
| CCNA2 | 2354.409023 | 22.22193738 | 61 | Cluster 1 | 43.43877551 |
| PLK1 | 1696.706116 | 12.22101703 | 60 | Cluster 1 | 43.43877551 |
| CCNB1 | 1556.017009 | 17.56934856 | 59 | Cluster 1 | 43.43877551 |
| FOXM1 | 3475.938241 | 37.85858264 | 59 | Cluster 1 | 42.50443262 |
| TOP2A | 1872.24983 | 14.29223362 | 58 | Cluster 1 | 43.43877551 |
| FGF2 | 8273.75708 | 20.885038 | 58 | Cluster 3 | 8.571428571 |
| MMP9 | 11157.23036 | 41.69827583 | 58 | Cluster 3 | 8.571428571 |
| AURKA | 1721.382186 | 21.10815952 | 57 | Cluster 1 | 43.43877551 |
| UBE2C | 1350.854127 | 8.797514964 | 56 | Cluster 1 | 43.43877551 |
| CDC20 | 319.7587412 | 4.765755878 | 53 | Cluster 1 | 43.43877551 |
| PBK | 1440.287098 | 24.82177476 | 53 | Cluster 1 | 43.43877551 |
| CDKN3 | 676.0546601 | 10.82879364 | 53 | Cluster 1 | 43.57092199 |
| BUB1B | 541.6261556 | 7.91732335 | 52 | Cluster 1 | 43.43877551 |
| CCNB2 | 176.484369 | 2.948688462 | 52 | Cluster 1 | 43.43877551 |
| KIF11 | 1109.132417 | 9.185848942 | 52 | Cluster 1 | 43.43877551 |
| TPX2 | 290.7348962 | 4.453869189 | 52 | Cluster 1 | 43.43877551 |
| BIRC5 | 1044.830253 | 19.93070049 | 52 | Cluster 1 | 43.43877551 |
| RRM2 | 1027.73984 | 10.84759185 | 52 | Cluster 1 | 43.43877551 |
| CEP55 | 1203.040881 | 20.73648306 | 52 | Cluster 1 | 43.43877551 |
| BUB1 | 726.2518548 | 7.162079605 | 51 | Cluster 1 | 43.43877551 |
| CDC6 | 48.7087622 | 0.847671883 | 51 | Cluster 1 | 43.43877551 |
| NUSAP1 | 331.6100849 | 4.368902836 | 51 | Cluster 1 | 43.43877551 |
| MYBL2 | 1273.978849 | 12.67920256 | 51 | Cluster 1 | 41.74545455 |
| DTL | 47.1127413 | 0.855992446 | 51 | Cluster 1 | 43.43877551 |
| KIAA0101 | 900.8900601 | 15.49074046 | 51 | Cluster 1 | 43.67437558 |
| TK1 | 1798.753096 | 22.5682644 | 51 | Cluster 1 | 42.75072464 |
| NEK2 | 549.1767657 | 8.859006829 | 50 | Cluster 1 | 41.57261795 |
| CDCA8 | 8.251854841 | 0.167060258 | 50 | Cluster 1 | 43.43877551 |
| DLGAP5 | 8.251854841 | 0.167060258 | 50 | Cluster 1 | 43.43877551 |
| PTTG1 | 444.5164766 | 8.66228168 | 50 | Cluster 1 | 43.43877551 |
| CENPF | 8.251854841 | 0.167060258 | 50 | Cluster 1 | 43.43877551 |
| KIF2C | 8.251854841 | 0.167060258 | 50 | Cluster 1 | 43.43877551 |
| KIF20A | 8.251854841 | 0.167060258 | 50 | Cluster 1 | 43.43877551 |
| KIF4A | 8.251854841 | 0.167060258 | 50 | Cluster 1 | 43.43877551 |
| MELK | 8.251854841 | 0.167060258 | 50 | Cluster 1 | 43.43877551 |
| SPAG5 | 8.251854841 | 0.167060258 | 50 | Cluster 1 | 43.43877551 |
| ASF1B | 50.98406498 | 0.916430515 | 50 | Cluster 1 | 42.64199815 |
| ZWINT | 5.420501941 | 0.115463925 | 49 | Cluster 1 | 43.43877551 |
| PRC1 | 168.9578578 | 3.111390823 | 49 | Cluster 1 | 43.67437558 |
| CDCA5 | 5.420501941 | 0.115463925 | 49 | Cluster 1 | 43.43877551 |
| NCAPH | 5.420501941 | 0.115463925 | 49 | Cluster 1 | 43.43877551 |
| NUF2 | 2.03658991 | 0.045557443 | 48 | Cluster 1 | 43.43877551 |
| COL1A1 | 4762.955949 | 24.58104326 | 48 | Cluster 3 | 8.192307692 |
| KPNA2 | 190.4541717 | 2.69718168 | 48 | Cluster 1 | 41.71594203 |
| ERBB2 | 6479.690068 | 35.4284731 | 48 | Cluster 6 | 5.300395257 |
| CKS2 | 1.812000997 | 0.042405948 | 47 | Cluster 1 | 43.57092199 |
| CENPU | 26.65741736 | 0.588158532 | 47 | Cluster 1 | 42.64199815 |
| UBE2T | 89.98380696 | 1.878968321 | 47 | Cluster 1 | 41.53376503 |
| PPARG | 6668.418003 | 32.92429669 | 46 | Cluster 6 | 5.323076923 |
| KIFC1 | 6.99586869 | 0.168253973 | 46 | Cluster 1 | 40.66868687 |
| FAM83D | 1.688490724 | 0.04132138 | 46 | Cluster 1 | 42.60222017 |
| LEP | 5986.293358 | 33.64406736 | 45 | Cluster 6 | 5.079365079 |
| ANLN | 0.833041782 | 0.02126051 | 45 | Cluster 1 | 41.83030303 |
| CXCL12 | 5036.000897 | 33.11289259 | 41 | Cluster 2 | 13 |
| LMNB1 | 736.4595647 | 10.97555541 | 38 | Cluster 1 | 35 |
| SPP1 | 3523.897413 | 44.00246485 | 37 | Cluster 4 | 7.35 |
| FOS | 4198.961124 | 59.2255398 | 35 | Cluster 3 | 9 |
| ADIPOQ | 2494.480954 | 26.09804751 | 34 | Cluster 3 | 9.230769231 |
| KRT19 | 3193.324096 | 31.85289474 | 34 | Cluster 4 | 7.125 |
| IQGAP3 | 300.7316401 | 12.69853275 | 34 |  | 31.942959 |
| EGR1 | 2954.86996 | 36.86800676 | 30 | Cluster 4 | 7.418181818 |
| LPL | 3942.112306 | 37.68852923 | 30 | Cluster 3 | 9.230769231 |
| BGN | 1088.750901 | 16.93980574 | 29 | Cluster 3 | 9 |
| KIT | 2722.385177 | 46.72248976 | 29 | Cluster 4 | 6.222222222 |
| CAV1 | 3914.160452 | 54.90390058 | 28 | Cluster 4 | 7.272727273 |
| POSTN | 3134.999119 | 45.55227311 | 28 | Cluster 3 | 8.836363636 |
| KRT14 | 2868.04843 | 38.07170538 | 27 | Cluster 3 | 7.846153846 |
| LIPE | 2146.001095 | 33.50106655 | 27 | Cluster 3 | 9.230769231 |
| MMP1 | 921.0954605 | 24.54722138 | 27 |  | 7.080882353 |
| FABP4 | 781.7368598 | 13.44188099 | 26 | Cluster 3 | 9.230769231 |
| MMP13 | 779.8681723 | 19.73564011 | 26 | Cluster 3 | 8.836363636 |
| KRT5 | 1640.100401 | 28.94607041 | 25 | Cluster 4 | 6.681318681 |
| KRT18 | 2430.495198 | 46.16592375 | 25 | Cluster 4 | 7.714285714 |
| SDC1 | 1702.234252 | 37.79818246 | 25 | Cluster 6 | 5.307692308 |
| CXCL10 | 983.9564044 | 23.9267582 | 24 | Cluster 2 | 13 |
| SLC2A4 | 1225.802446 | 18.34390074 | 24 | Cluster 3 | 9.696969697 |
| ANXA1 | 1372.975642 | 41.83066434 | 24 | Cluster 2 | 13 |
| CXCL2 | 1063.098286 | 21.809933 | 22 | Cluster 2 | 13 |
| MUC1 | 1772.742262 | 37.06833779 | 22 | Cluster 4 | 6.974358974 |
| KRT8 | 774.4640983 | 29.75722232 | 21 | Cluster 4 | 7.714285714 |
| SAA1 | 1118.576833 | 32.5463693 | 21 | Cluster 2 | 13 |
| PLIN1 | 577.336648 | 17.32543998 | 20 | Cluster 3 | 9.487179487 |
| FOXA1 | 1826.747611 | 32.46513173 | 20 | Cluster 4 | 7 |
| KLF4 | 824.8297442 | 38.46494217 | 20 | Cluster 6 | 5.357142857 |

**Abbreviation:** PPI: protein-protein interaction.
