# Supplementary material for: 6-lncRNA Assessment Model for Monitoring and Prognosis of HER2-Positive Breast Cancer: Based on Transcriptome Data
Source: Pathol Oncol Res. 2021 Apr 13;27:609083. doi: 10.3389/pore.2021.609083 (PMC8262145; doi:10.3389/pore.2021.609083)
Supplement: Supplementary file 1 [file DataSheet1.ZIP › Supplementary materials/Table S5.doc]

**Table S5 The relationship between the expression of the core lncRNAs and different clinical parameters in HER2-positive breast cancer.**

| lncRNA | Gender-t | Gender-p | Stage-t | Stage-p | Therapy-t | Therapy-p |
| --- | --- | --- | --- | --- | --- | --- |
| MIR762HG | 2.387721369 | 0.042240281 | 0.618017572 | 0.542217701 | 0.513868669 | 0.619119697 |
| AC069277.1 | 3.951508998 | 0.000137394 | -0.634172717 | 0.534120213 | 1.300807086 | 0.226145971 |
| AC046168.1 | 2.646622451 | 0.009351746 | -0.308459466 | 0.761279267 | -0.472215286 | 0.640535871 |
| LINC00528 | 1.465220018 | 0.183570858 | 1.930803441 | 0.060616568 | -2.327168094 | 0.026012645 |
| AP000904.1 | 2.372340281 | 0.03342122 | -0.591058754 | 0.560201423 | 1.429969176 | 0.188311612 |
| AC010595.1 | 3.156837613 | 0.003747776 | 0.920391984 | 0.363430882 | 1.275297375 | 0.235082279 |
